# The paeonol target gene autophagy-related 5 has a potential therapeutic value in psoriasis treatment

**DOI:** 10.7717/peerj.11278

**Published:** 2021-05-25

**Authors:** Qian Zhang, Hongqiao Shi, Jiaan Zhang, Chenxue Jiang, Chunxiang Zhou

**Affiliations:** 1 Department of Dermatology, Nanjing Hospital of Chinese Medicine Affiliated to Nanjing University of Chinese Medicine, Nanjing, Jiangsu Province, China; 2 Institute of Dermatology, Jiangsu Key Laboratory of Molecular Biology for Skin Diseases and STIs,Chinese Academy of Medical Science&Peking Union Medical College, Nanjing, Jiangsu Province, China; 3 School of Foreign Languages, Nanjing University of Chinese Medicine, Nanjing, Jiangsu Province, China; 4 College of Traditional Chinese Medicine and College of Integrated Chinese and Western Medicine, Nanjing, Jiangsu Province, China

**Keywords:** Autophagy related 5, Psoriasis, Differentially expressed genes, Autophagy, Paeonol

## Abstract

**Background:**

Paeonol is a potent therapy for psoriasis. This study aimed to screen out paeonol-targeted genes in psoriasis and validate the potential of using paeonol for the management of psoriasis.

**Methods:**

Microarray datasets were obtained from the Gene Expression Omnibus. The differentially expressed genes (DEGs) in the lesional skin samples and the overlapping genes between DEGs and paeonol- and psoriasis-related genes were defined as potential targets for psoriasis. After being treated with si-ATG5 and pc-ATG5, human HaCaT cells were treated with 100 ng/ml IL-22 and 10 ng/ml TNF-*α* with and without paeonol. Cell proliferation, apoptosis, and expression of interleukin (IL)-6, IL-1*β*, Beclin 1, ATG5, and p62 in HaCaT cells were determined using ESLIA, PCR, and Western blot analysis.

**Results:**

A total of 779 DEGs were identified in the lesional skin samples compared with the non-lesional tissues. The autophagy-related 5 (*ATG5*) gene was the only gene that overlapped between the DEGs and genes related to paeonol and psoriasis. Cell proliferation, inflammatory cytokines (IL-6 and IL-1*β*), and ATG5 expression were increased in IL-22/TNF-*α*-stimulated HaCaT (model) cells compared with control. Paeonol treatment rescued all changes. si-ATG5 transfection increased inflammation and apoptosis in model cells compared with controls. pc-ATG5 prevented IL-22/TNF-*α*-induced changes in HaCaT cells. Also, si-ATG5 decreased p62 and Beclin 1 proteins, while pc-ATG5 increased them both.

**Conclusions:**

*ATG5*-dependent autophagy plays a crucial role in psoriasis. The *ATG5* gene might be a therapeutic target for the management of in vitro psoriasis.

## Introduction

Psoriasis is a common skin lesion condition characterized by aberrant proliferation of keratinocytes and chronic inflammation (Boehncke & Schön, 2015). This disorder influences 2–4% of the general population and almost 70% of patients diagnosed with psoriasis before age 40 (De Simone et al., 2019). The potential pathogenesis of psoriasis was complicated and multifactorial. Specifically, the genetic factors, infection, endocrine disorder, environmental and mental elements might all contribute to the initiation of psoriasis (Menter et al., 2008).

Psoriasis is thought to result from genetic and environmental influences. In recent decades, numerous characteristic markers concerned with psoriasis have been identified by bioinformatics analysis, which facilitates early recognition and targeted treatment for psoriasis. Zhang et al. (2019) identified that interferon-*α* (INF-*α*)-inducible genes were the major feature in scalp psoriasis. Xie et al. (2014) carried out a microarray analysis to extract possible bio-targets in the molecular pathogenesis of psoriasis and they found that 10 genes were mainly responsible for the defense response pathway and for distinguishing the psoriasis lesions from non-lesions (Xie et al., 2014). Melero et al (2018) pointed out that many cell cycle-related genes were up-regulated in psoriasis samples according to bioinformatics analysis. Notably, Mei & Mei (2017) performed a meta-integrated investigation based on four datasets of psoriasis and they revealed that 70 genes were considered as potential treatment targets for psoriasis.

Several signaling pathways including the nuclear factor *κ*B (NF-*κ*B), Mitogen-activated protein kinase (MAPK), mammalian target of rapamycin (mTOR), and tumor necrosis factor *α* (TNF-*α*) play crucial roles in the onset of psoriasis (Jin et al., 2016; Zhai et al., 2017; Zhao et al., 2015). Besides, most of them could be used as the targets of paeonol for the management of psoriasis and interleukin-induced inflammation (Meng et al., 2017; Lou et al., 2017; Zhai et al., 2017). Paeonol is a major phenolic component of *Moutan Cortex* which has a wide range of bioactivities including antioxidant, anti-inflammatory, cardioprotective, and neuroprotective (Ding et al., 2016; Gao et al., 2019; Liu et al., 2019). It is widely used to suppress oxidant and inflammatory conditions (Jin et al., 2016; Zhai et al., 2017). Besides, paeonol has a bidirectional effect on autophagy that is recently identified to be correlated with psoriasis (Gao et al., 2019a; Li et al., 2018). Paeonol induces cytoprotective autophagy in ovarian cancer cells (Gao et al., 2019a) and also inhibits autophagy in vascular endothelial cells (Li et al., 2018). However, the precise mechanism through which paeonol affects psoriasis is poorly understood.

We performed this integrated bioinformatics analysis to select the potential target of paeonol for the management of psoriasis. Four gene expression profiles were downloaded to select the differentially expressed genes (DEGs) in the lesional psoriatic skin. Those DEGs related to paeonol were identified. Cellular experiments were performed to validate the potential of using the paeonol-targeted gene for the management of psoriasis.

## Material and Methods

### Data source and pre-processing

**Figure 1:**
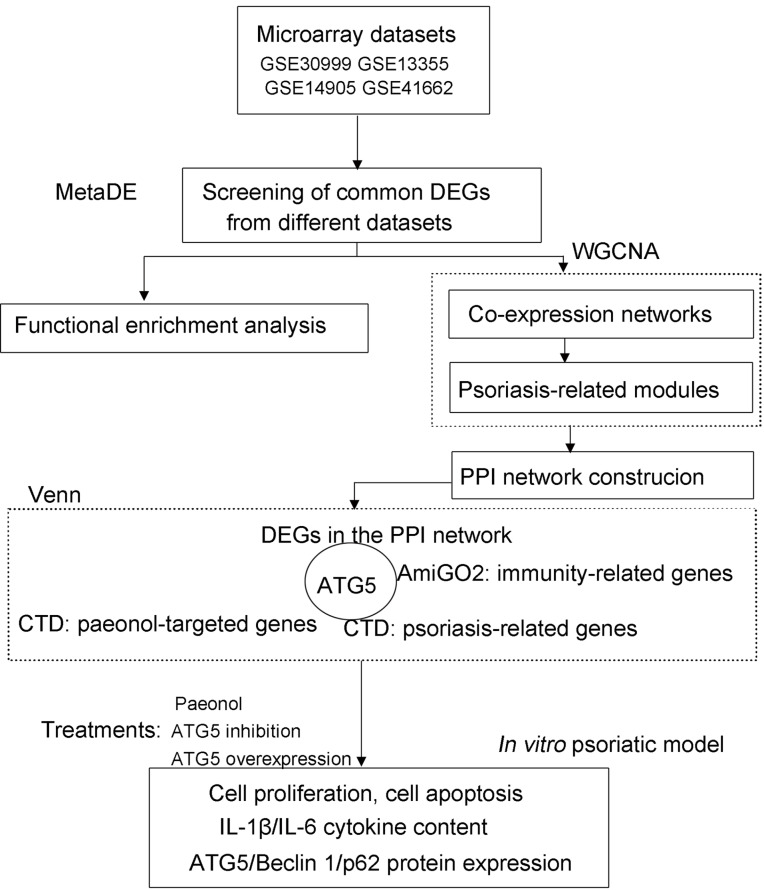
The flow chart of data analysis in this study. CTD, Comparative Toxicogenomics Database; DEGs, differentially expressed genes; WGCNA, weighted gene co-expression network analysis.

The Gene Expression Omnibus (GEO; http://www.ncbi.nlm.nih.gov/geo/) (Clough, 2016) repository was retrieved using the keywords of “psoriasis”, “lesion”, and “homo sapiens” to obtain microarray datasets from psoriasis patients. The eligible datasets (GSE30999, GSE13355, GSE14905, and GSE41662; Affymetrix platform; Table S1) were selected following screening criteria: (1) microarray expression profile from skin tissue of patients undergoing psoriasis; (2) ≥ 40 samples of lesion and non-lesion cases. The flow chart of data analysis in this study is shown in Fig. 1.

### DEGs identification by meta-analysis

We employed the MetaQC package (https://cran.r-project.org/web/packages/MetaQC/index.html) in R 3.4.1 to implement quality control (QC). The principal component analysis and standardized mean rank score were also used to evaluate and screen data information. The DEGs were extracted using MetaDE.ES in MetaDE (Chang, Sibille & Tseng, 2013) package (https://cran.r-project.org/web/packages/MetaDE). The cutoffs of consistently DEGs identification were set as tau^2^ = 0 and Q pval > 0.05 of heterogeneity test, false discovery rate (FDR) < 0.05 and —log_2_fold change (FC)— > 0.263. Functional analyses of these DEGs, including the Gene Ontology (GO) biological process and Kyoto Encyclopedia of Genes and Genomes (KEGG) pathway, were performed by DAVID (Huangda, Sherman & Lempicki, 2009) (version 6.8; https://david.ncifcrf.gov/). *P* value < 0.05 was used as the threshold of significant enrichment.

### Screening of module DEGs related to psoriasis

Weighted gene co-expression network analysis (WGCNA) (Chen et al., 2012) has widely applied to identify the gene module associated with diseases. GSE30999 was set as the training dataset while other thee datasets (GSE13355, GSE14905, and GSE41662) served as the validation datasets. The stable gene module linked with disease features was screened using WGCNA software (version 1.61; https://cran.r-project.org/web/packages/WGCNA/index.html) with the thresholds of gene number ≥ 25, cutHeight = 0.995, preservation *z* score >5, and correlation >0.6.

### Protein-protein interaction (PPI) network construction

The STRING (Szklarczyk et al., 2017) database (version 10.5; https://string-db.org/) was used to examine the protein product interactions of genes in the module. The PPI network was constructed and visualized with Cytoscape (Shannon et al., 2003) (version 3.6.1; http://www.cytoscape.org/).

### Selecting in database

The genes and pathways related to psoriasis and paeonol were selected from the Comparative Toxicogenomics Database (CTD, 2020 update; http://ctd.mdibl.org/). The immunity-related genes were selected from the AmiGO 2 (http://amigo.geneontology.org/amigo). The overlapping genes between the DEGs and the genes in the related pathways were selected and regarded as the target of further experiments. The MCODE plugin (http://apps.cytoscape.org/apps/mcode) was used to identify modules in the PPI network.

### Cell line, culture conditions, treatment, and psoriatic model

Human immortalized keratinocyte HaCaT cell line (Central Laboratory of Peking University Third Hospital, Beijing, China) were maintained in complete Dulbecco’s modified Eagle’s medium (DMEM; Hyclone, Thermo Scientific, Epsom, UK). To mimic psoriatic conditions, HaCaT cells (1 ×10^6^cells/ml) were cultured in complete DMEM for 12 h in 6-well plates, followed with incubation in serum-free DMEM supplemented with 100 ng/ml IL-22 and 10 ng/ml TNF-*α* (PeproTech House, London, UK) for 24 h (Wu et al., 2020), with or without paeonol (National Institutes for Food and Drug Control, Beijing, China; 40 µg/ml) (Meng et al., 2017; Quan, 2019). All cells were incubated at 37 °C 5% CO_2_.

### Plasmid construction and cell transfection

The full-length coding sequence (CDS) of the human autophagy-related 5 (ATG5) gene was multiplied by PCR using the ATG5 specific primers with *Bam* HI/*Xho* I restriction enzyme sites (Table 1). The plasmid pc-ATG5 was constructed by cloning the PCR products into the pcDNA3.1 vectors (Genechem Co. Ltd, Shanghai, China). The short interfering RNAs (siRNA) targeting ATG5 (si-ATG5) and scramble sequences were purchased from the Genechem Co. Ltd. HaCaT cells (1 × 10^5^ cells/well) were transfected with si-ATG5, pc-ATG5, scramble sequences, and pcDNA3.1 vector for 24 h. Cell transfection was performed in triplicate using Lipofectamine 2000 regents (Invitrogen, Carlsbad, CA, USA). Psoriatic HaCaT cells (1 × 10^5^ cells/well) were stimulated with 100 ng/ml IL-22 and 10 ng/ml TNF-*α* for 24 h, with or without paeonol treatment.

**Table 1:** The sequences of primers used in this study.

Gene	Use	Primer	Sequence (5′-3′)	Product length
ATG5	PCR	Forward	5′-ATGACAGATGACAAAGATG-3′	182 bp
		Reverse	5′-CTCATAACCTTCTGAAAGTG-3′	
ATG5	Clone	Forward	5′-CGGGATCC(*Bam* HI) ATGACAGATGACAAAGATGTGC-3′	820 bp
		Reverse	5′-CCG CTCGAG (*Xho* I) TCAATCTGTTGGCTGTGGGATG-3′	
IL-6	PCR	Forward	5′-TAGTGAGGAACAAGCCAGAGC-3′	104 bp
		Reverse	5′-TTGGGTCAGGGGTGGTTATTG-3′	
IL-1*β*	PCR	Forward	5′-CAGCCAATCTTCATTGCTCAAG-3′	105 bp
		Reverse	5′-GAACAAGTCATCCTCATTGCC-3′	
GAPDH	PCR	Forward	5′-CATGAGAAGTATGACAACAGCCT-3′	113 bp
		Reverse	5′-AGTCCTTCCACGATACCAAAGT-3′	

### Proliferation assay

Psoriatic HaCaT cells were treated with trypsin at 12 h and 24 h post-treatment. Cell Counting Kit-8 (CCK8; Dojindo, Japan) solution was added into cell culture and incubated for 2 h. Cell viability at 450 nm absorbance was analyzed using a microplate reader (Thermo Labsystems, Helsinki, Finland).

### Cell apoptosis assay

Cell apoptosis was analyzed using flow cytometry and an annexin V-Cy5-labeled Apoptosis Detection Kit (Beyotime Institute of Biotechnology, Nanjing, China). Briefly, HaCaT cells were harvested and were then suspended in 5 µl of Annexin V-Cy5 and 5 µl of PI. A FACS Calibur flow cytometer (BD Biosciences, Franklin Lakes, NJ, USA) was used for apoptosis analysis.

### Measurement of cytokines

The profiles of IL-1*β* and IL-6 in cell culture were detected using the enzyme-linked immunosorbent assay (ELISA) and commercial ELISA kits (Cusabio Biotech Corporation, USA). A microplate reader (Thermo Labsystems) was used for data analysis.

### Protein extraction and Western blot analysis

After treatment for 24 h, HaCaT cells were lysed in triplicate using RIPA buffer (Beyotime Institute of Biotechnology, Beijing, China). Protein concentration was determined using a BCA protein assay kit (Thermo Fisher Scientific, Inc., Waltham, USA). Proteins (30 µg) were separated using 10% SDS-PAGE and were transferred onto the polyvinylidene fluoride (PVDF) membranes (Millipore, Billerica, MA, USA). Membranes were blocked using 5% skim milk (Beyotime) and incubated with the first primary antibodies including anti-ATG5 (1: 1000, Abcam), anti-Beclin 1 (1: 1500, Abcam), anti-p62 (1: 1000, Abcam), and anti-GAPDH (1: 10000, Abcam) at 4 °C overnight. The secondary incubation with HRP goat anti-rabbit IgG (1: 20000, Boster Biotechnology, Wuhan, China) was conducted at room temperature for 1 h. Protein bands were analyzed using the enhanced chemiluminescence (ECL) system.

### RNA isolation and quantitative real-time PCR

Total RNA was extracted from psoriatic HaCaT cells using TRIzol Reagent (Invitrogen) at 24 h after stimulation or transfection. The RNA was reversely transcribed to cDNA using a PrimeScript RT-polymerase kit (TaKaRa, Dalian, China) to the manufacturer’s protocol.

The expression levels of genes were detected using an SYBR ExScript qRT-PCR Kit (TaKaRa). GAPDH was used as the internal control. Gene-specific PCR primer pairs were synthesized by Sangon (Shanghai, China; Table 1). PCR amplification was conducted according to the following reaction conditions: 95 °C for 5 min; 38 cycles of 95 °C for 30 s, 60 °C for 40 s, and 72 °C for 45 s. The relative expression levels of genes were analyzed using the 2-^△△Ct^ methods.

### Statistical analysis

Each experiment was performed in triplicate in this study. Statistical analysis was performed using the GraphPad Prism 8.0 software (GraphPad Prism Inc., La Jolla, CA, USA). All data were expressed as mean ± standard deviation. Data differences across groups were analyzed using the ordinary one-way analysis of variance (ANOVA) test followed by the Tukey test as post hoc. *P* values <  0.05 were considered statistically significant.

## Results

### Identification of consistently DEGs and functional analyses

**Figure 2:**
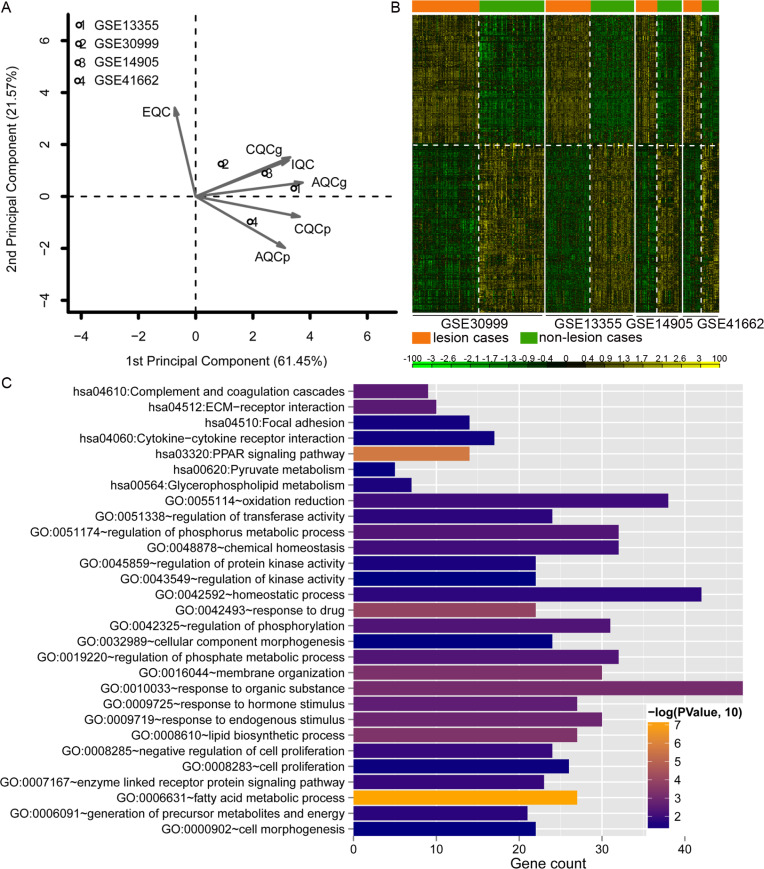
Analysis of dataset quality, clustering and enrichment analysis of the differentially expressed genes (DEGs). (A) The principal component result of the four datasets. The vertical axis showed the first principal component and horizontal axis indicated the second principal component. IQC, internal quality control; EQC, external quality control; CQCg, consistency quality control, genes; CQCp: consistency quality control, pathways; AQCg, accuracy quality control, genes; AQCp, accuracy quality control genes, pathways. (B) The heatmap clustering of the 779 consistently DEGs across these four datasets. (C) the functional enrichment analyses of the consistently DEGs. The vertical axis showed the number of genes and horizontal axis denotes the name of enrichment terms or pathways. The color of bar represents the significance.

Data analysis showed that four datasets exhibited even distribution and good quality (Fig. 2A). A total of 779 DEGs were identified (Table S1). The heatmap clustering of the DEGs in samples is shown in Fig. 2B. Functional enrichment analysis indicated that these genes were enriched with GO biological processes associated with regulation of phosphorus metabolic process (GO:0051174), negative regulation of cell proliferation (GO:0008285), and lipid biosynthetic process (GO:0008610; Fig. 2C), and KEGG pathways including PPAR signaling pathway (hsa03320), complement and coagulation cascades (hsa04610), and ECM-receptor interaction pathway (hsa04512; Fig. 2C).

**Figure 3:**
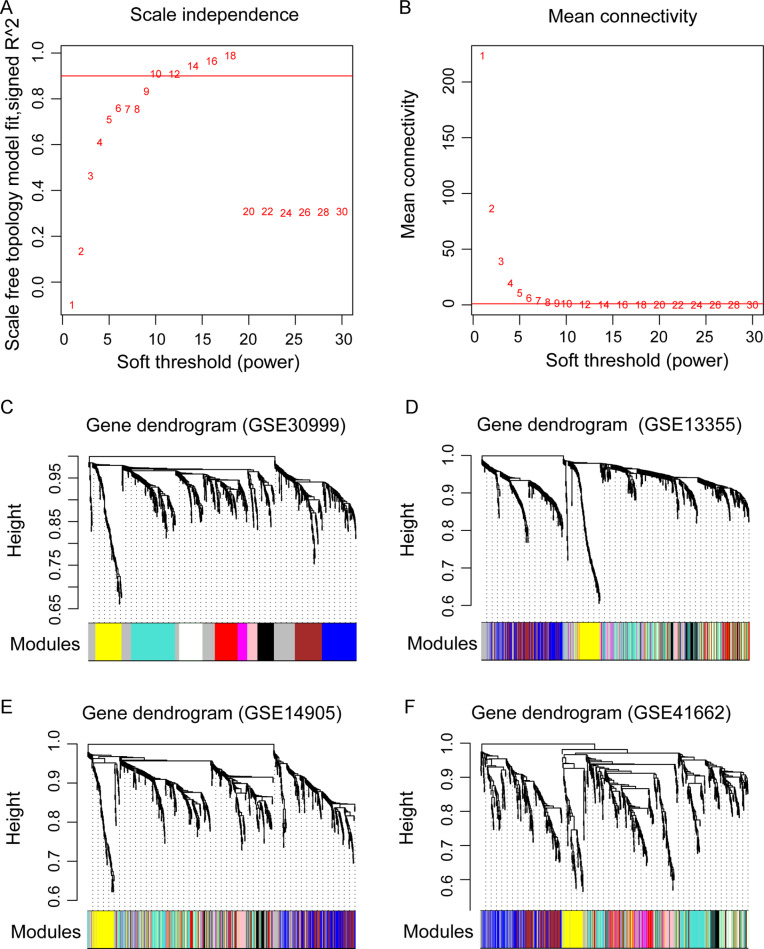
The weighted gene co-expression network analysis (WGCNA) of differentially expressed genes in the four datasets. (A) The diagram of matrix weight parameter power selection. The *x* axis represents the power of the weighting parameter and the *y* axis represents the scale-free topology model fit signed *R*^2^ in the network. (B) The diagram of gene connectivity under different power parameters. The red line indicates the value under different power parameter and the average node connectivity of 1. (C–F) the WGCNA module in the four datasets. The GSE30999 dataset is used as the training dataset, while the other three were used as the validation datasets. The eight modules are represented by different colors.

### WGCNA extraction of gene modules related to psoriasis

**Figure 4:**
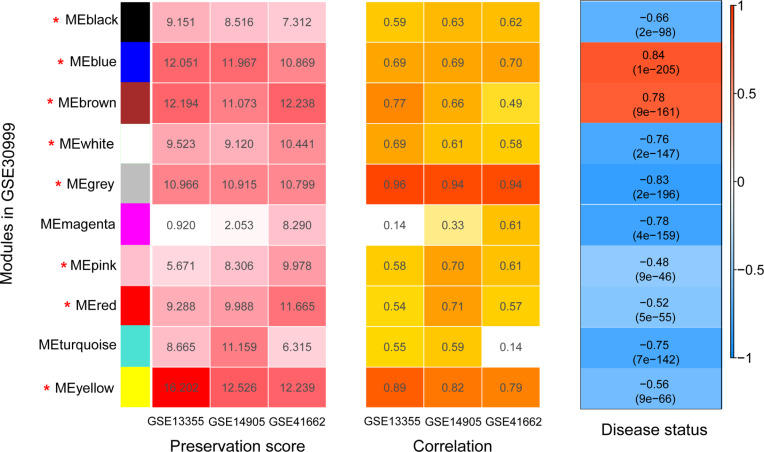
The heatmap indicating the correlation of modules with psoriasis status. The modules were identified using weighted gene co-expression network analysis (WGCNA) based on the differentially expressed genes. Module has a positive and negative correlation with psoriasis status is represented by red and blue color, respectively. *P* < 0.05 is the cutoff of significant correlation.

Before WGCNA, we found that the gene expression profiles in four datasets had high and positive correlations and connectivity (Figs. S1A and S1B). The soft-thresholding power = 10 when the square of the correlation coefficient = 0.9 and mean connectivity =1 (Figs. 3A–3B). Ten WGCNA modules were identified in the training dataset (Fig. 3C) and the validation datasets (GSE13355, GSE14905, and GSE41662; Figs. 3D–3F). Module preservation analysis showed that eight modules related to psoriasis traits. Four modules, including the black, white, red, and turquoise, had negative correlations with the psoriatic phenotype (preservation *z* score >5, correlation <−0.6, and *p* < 0.05, Fig. 4) and two modules, including blue (98 upregulated DEGs) and brown (79 upregulated DEGs) modules, had positive correlations with psoriasis (preservation z score >5 and correlation >0.6, Fig. 4).

### PPI network construction

The PPI network was constructed using the 177 upregulated DEGs in the blue and brown modules. Accordingly, the PPI network consisted of 236 edges (interaction pairs) and 133 nodes (upregulated gene products; Fig. S2). These genes enriched in 31 biological processes, including cell cycle (GO:0007049) and apoptosis (GO:0006915; Table S2), and 12 KEGG pathways including Cell cycle (hsa04110), Natural killer cell mediated cytotoxicity (hsa04650), Cytokine-cytokine receptor interaction (hsa04060), and T cell receptor signaling pathway (hsa04660; Table 2). The 133 upregulated genes are listed in Table S3.

**Table 2:** The Kyoto Encyclopedia of Genes and Genomes pathways associated with the differentially expressed genes in the protein-protein interaction network.

**Term**	**Count**	*P*Value	**Genes**
hsa00100:Steroid biosynthesis	2	1.40E−02	CYP51A1, SQLE
hsa04662:B cell receptor signaling pathway	3	1.42E−02	BCL10, LYN, SYK
hsa04664:Fc epsilon RI signaling pathway	3	1.51E−02	LYN, LCP2, SYK
hsa04630:Jak-STAT signaling pathway	4	1.57E−02	TSLP, IL10RA, IL12B, IL7R
hsa04666:Fc gamma R-mediated phagocytosis	3	2.05E−02	PLD2, LYN, SYK
hsa04660:T cell receptor signaling pathway	3	2.48E−02	BCL10, ITK, LCP2
hsa03030:DNA replication	2	2.75E−02	RNASEH1, MCM6
hsa04110:Cell cycle	3	3.04E−02	PKMYT1, CDK7, MCM6
hsa00190:Oxidative phosphorylation	3	3.20E−02	UQCR10, COX10, COX5A
hsa04650:Natural killer cell mediated cytotoxicity	3	3.30E−02	FCGR3B, LCP2, SYK
hsa04060:Cytokine-cytokine receptor interaction	4	4.11E−02	TSLP, IL10RA, IL12B, IL7R
hsa04622:RIG-I-like receptor signaling pathway	2	4.70E−02	ATG5, IL12B

### Selection of psoriasis-related and paeonol-targeted genes

To select the genes that have an important role in psoriasis, genes associated with immunity and psoriasis were identified from the 133 DEGs. A total of 119 DEGs (Table S3) were included in the lists of immunity-related genes (*n* = 3,324) and psoriasis-related genes (17,246). Also, nine common pathways related to psoriasis, paeonol, and DEGs were identified from the CTD, including the Jak-STAT signaling pathway (hsa04630), Cytokine-cytokine receptor interaction (hsa04060), and RIG-I-like receptor signaling pathway (hsa04622; Table 3). Only two genes (ATG5 and IL12B) were overlapped between the DEGs and genes in the CTD database.

**Table 3:** The overlapping Kyoto Encyclopedia of Genes and Genomes (KEGG) pathways associated with the differentially expressed genes in the protein–protein interaction network.

**Term**	**Count**	*P*-value	**Genes in KEGG**	**Genes in CTD**
hsa04662:B cell receptor signaling pathway	3	0.014	BCL10, LYN, SYK	
hsa04664:Fc epsilon RI signaling pathway	3	0.015	LYN, LCP2, SYK	
hsa04630:Jak-STAT signaling pathway	4	0.016	TSLP, IL10RA, IL12B, IL7R	IL12B
hsa04660:T cell receptor signaling pathway	3	0.025	BCL10, ITK, LCP2	
hsa03030:DNA replication	2	0.028	RNASEH1, MCM6	
sa04110:Cell cycle	3	0.030	PKMYT1, CDK7, MCM6	
hsa04650:Natural killer cell mediated cytotoxicity	3	0.033	FCGR3B, LCP2, SYK	
hsa04060:Cytokine-cytokine receptor interaction	4	0.041	TSLP, IL10RA, IL12B, IL7R	IL12B
hsa04622:RIG-I-like receptor signaling pathway	2	0.047	ATG5, IL12B	ATG5, IL12B

**Notes.**

CTD, Comparative Toxicogenomics Database. The overlapping genes associated with psoriasis in the CTD.

**Figure 5:**
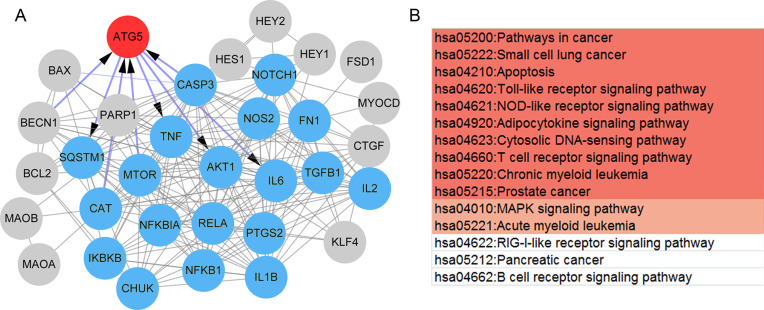
The protein-protein interaction (PPI) network and pathways of the paeonol-related genes. (A) The PPI network of the 35 paeonol-targeted genes in the Comparative Toxicogenomics Database. ATG5 (red) is the only common gene between paeonol-targeted genes and the 119 immunity and psoriasis-related genes genes. The MCODE module consisted of 19 nodes (Blue). (B) The pathways associated with the 19 nodes. Color notes the significance level (from high to low).

Also, a total of 35 paeonol-targeted genes were identified from the CTD database and the network of paeonol-targeted genes is shown in Fig. 5A. ATG5 was the only overlapped gene between paeonol-targeted genes and the DEGs (Fig. 5A). ATG5 interplayed with IL-6, CASP3, TNF, Akt1, and mTOR (Fig. 5A). Besides, we identified an MCODE module (score=15.73) consisting of 19 genes including IL-6, CASP3, TNF, Akt1, and mTOR. This module was related to 15 pathways including Apoptosis (hsa04210), Toll-like receptor signaling pathway (hsa04620), MAPK signaling pathway (hsa04010), and RIG-I-like receptor signaling pathway (hsa04622; Fig. 5B). These results indicated that the ATG5 gene might have an important role in psoriasis and might be a target of paeonol.

### Paeonol suppresses psoriasis

**Figure 6:**
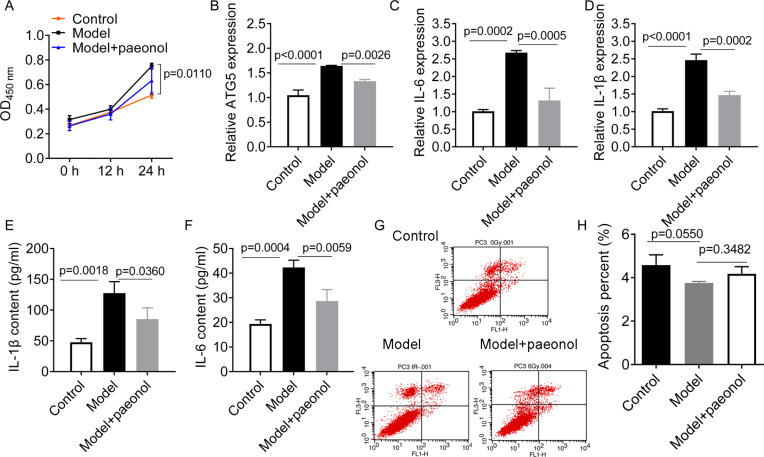
The effect of paeonol on the cellular psoriatic model (HaCaT cells). (A) The result of cell viability assay. (B) The relative expression level of ATG5 gene in HaCaT cells. (C and D), The relative expression levels of IL-6 and IL-1*β* mRNAs in HaCaT cells. PCR analysis was used for the analysis of gene expression levels. (E and F) The contents of IL-6 and IL-1*β* cytokines in HaCaT cells. ELISA assay was performed for the measurement of cytokines’ content. G and H, the cell apoptosis assay by flow cytometry. Data are expressed as mean ±  standard deviation. The differences were analyzed using the ordinary one-way ANOVA test followed by Tukey test.

To investigate the influence of ATG5 and paeonol intervention on psoriasis, we established an in vitro psoriatic model using IL-22/TNF-*α* stimuli and treated it with paeonol. IL-22/TNF-*α* stimuli increased HaCaT cell viability (*p* = 0.0110; Fig. 6A), ATG5 expression (*p* < 0.0001, Fig. 6B), and the expression of IL-6 and IL-1*β* (*p* < 0.01; Figs. 6C–6F). However, paeonol significantly reversed IL-22/TNF-*α*-induced changes in HaCaT cells. Paeonol decreased ATG5 expression (*p* = 0.0026) and the expression of IL-6 and IL-1*β* in IL-22/TNF- *α*-treated HaCaT cells (*p* < 0.05; Figs. 6C–6F). We also found that IL-22/TNF-*α* stimuli induced less obvious reduction in the apoptotic percentage of HaCaT cells, from 4.58 ± 0.47% in control cells to 3.36 ± 0.07% in model cells (IL-22/TNF- *α*-treated HaCaT cells; *p* = 0.0550, Figs. 6G and 6H). The effect of paeonol on apoptosis was not evident (*p* = 0.3482; Fig. 6G and 6H). These results might indicate that paeonol has an inhibitory effect on psoriasis.

### Si-ATG5 increases IL-6 and IL-1*β* and promotes apoptosis

**Figure 7:**
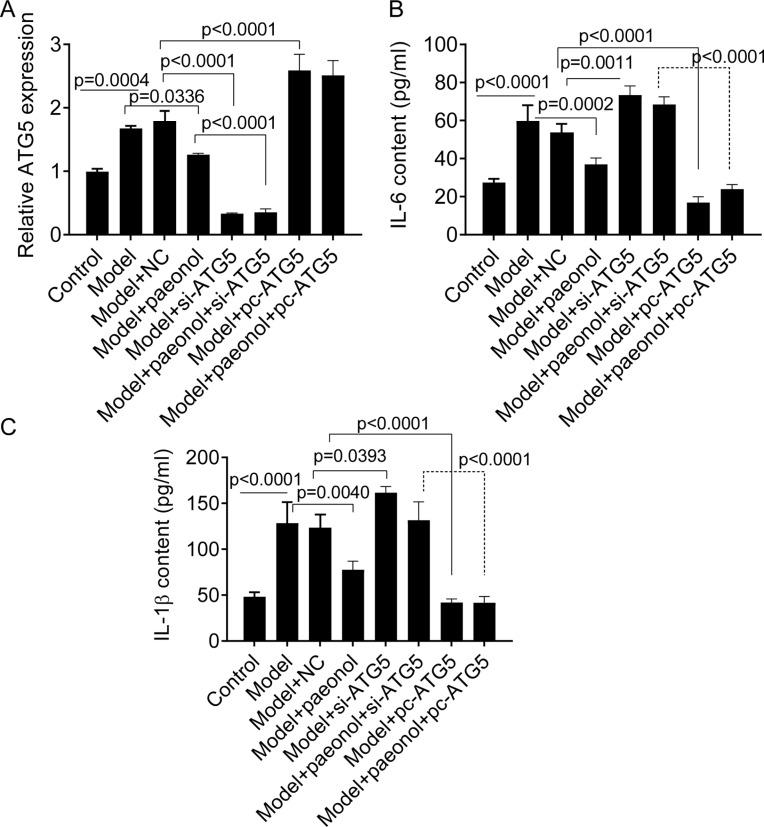
The effect of ATG5 expression and paeonol on inflammation in HaCaT cell apoptosis. (A) The expression level of ATG5 mRNA by PCR assay. (B and C) The production of cellular IL-6 and IL-1*β* by ELISA assay. Data are expressed as mean ± standard deviation. The differences were analyzed using the ordinary one-way ANOVA test followed by Tukey test.

We transfected si-ATG5 and pc-ATG5 into HaCaT cells and detected the expression of IL-6 and IL-1*β* to investigate whether the ATG5 gene could be used as a therapeutic target for the management of psoriasis. PCR analysis confirmed that si-ATG5 and pc-ATG5 transfection significantly decreased and increased the expression level of ATG5 in model cells, respectively (*p* < 0.0001; Fig. 7A). Also, si-ATG5 dramatically increased the contents of IL-6 and IL-1 *β* in model cells compared with negative controls (NC; *p* = 0.0011 for IL-6, and *p* = 0.0393 for IL-1*β*; Figs. 7B and 7C). However, we observed that pc-ATG5 transfection significantly decreased the contents of IL-6 and IL-1*β* in model cells compared with NC (Fig. 7B and 7C).

**Figure 8:**
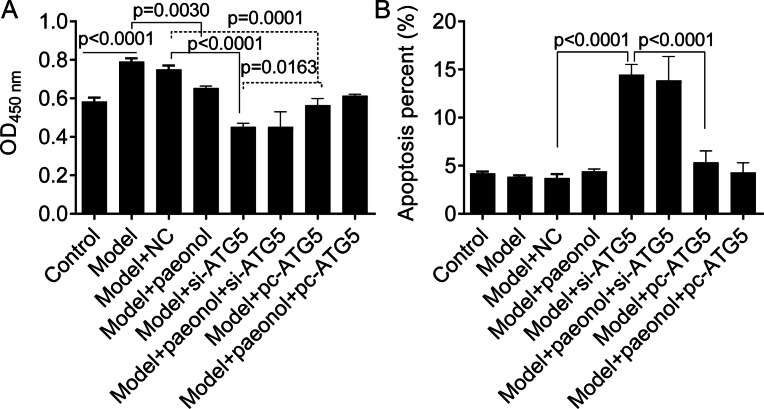
The effect of ATG5 expression and paeonol on HaCaT cell proliferation and apoptosis. (A) The cell viability by the CCK8 assay. (B) The apoptosis of HaCaT cells assessed by flow cytometry. Data are expressed as mean ± standard deviation. The differences were analyzed using the ordinary one-way ANOVA test followed by Tukey test.

Besides, we found si-ATG5 significantly decreased HaCaT cell viability (*p* < 0.0001; Fig. 8A) and increased the apoptotic percentage of model cells (from 3.84 ± 0.15% to 14.45 ± 1.07%, *p* < 0.0001; Fig. 8B). However, pc-ATG5 transfection prevented the effects of IL-22/TNF-*α* stimuli on HaCaT cell viability and apoptosis (Figs. 8A and 8B). These results showed that ATG5 expression played crucial roles in controlling inflammation and cell viability in HaCaT cell apoptosis.

### Expression of Beclin 1 and p62

**Figure 9:**
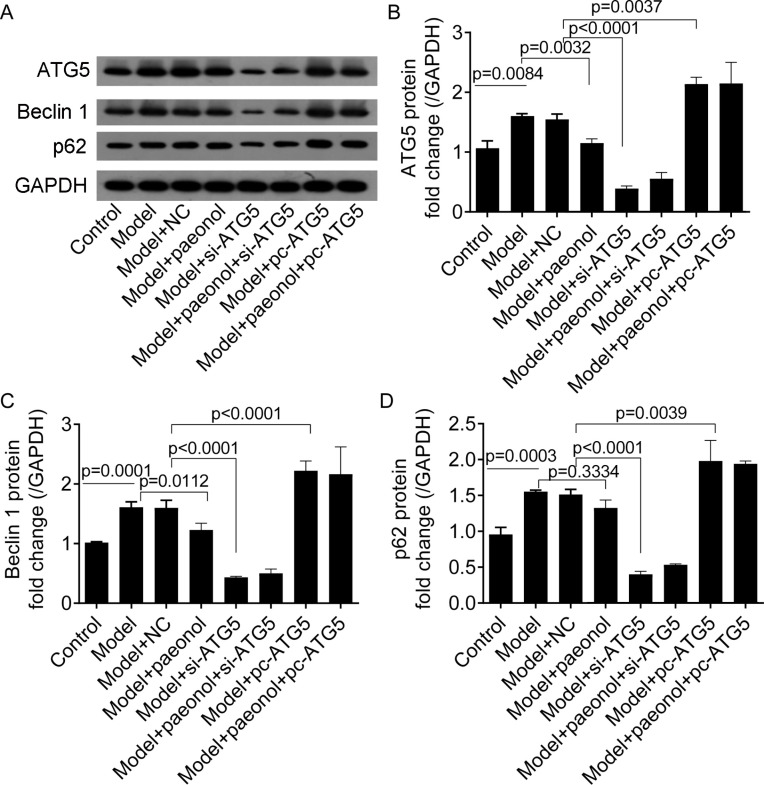
The expression of ATG5, Beclin 1, p62, and GAPDH proteins in HaCaT cells. (A) The protein bands of of ATG5 (32 kDa), Beclin 1 (52 kDa), p62 (62 kDa), and GAPDH (36 kDa) proteins. (B–D) The fold changes of ATG5, Beclin 1 and p62 proteins in HaCaT cells under different treatments. Data are expressed as mean ± standard deviation. The differences were analyzed using the ordinary one-way ANOVA test followed by Tukey test.

To investigate the effect of ATG5 expression on autophagy in HaCaT cells, we detected the expression levels of two autophagy-related proteins, including Beclin 1 and p62. Western blot analysis showed that IL-22/TNF-*α* stimuli significantly increased the fold changes of ATG5, Beclin 1, and p62 proteins compared with control cells (*p* < 0.001; Figs. 9A–9D). Paeonol significantly decreased the expression of ATG5 and Beclin 1 proteins in HaCaT cells (*p* < 0.05; Figs. 9A–9C) but not p62 ( *p* = 0.3334; Fig. 9D). Besides, si-ATG5 transfection decreased the expression of Beclin 1, ATG5, and p62 proteins compared with NC (*p* < 0.0001; Figs. 9A–9D) and pc-ATG5 transfection increased Beclin 1, ATG5, and p62 proteins (*p* < 0.01; Figs. 9A–9D), respectively. However, the effects of paeonol on the expression of Beclin 1, ATG5, and p62 proteins in HaCaT cells transfected with si-ATG5 or pc-ATG5 were no evident.

## Discussion

Studies focusing on illuminating psoriasis pathogenesis using bioinformatics methods have been performed over the past few years (Mei, 2017; Zhang et al., 2019). Zhang et al. (2019) identified that INF-*α*-inducible genes are the characteristic genes in scalp psoriasis. Also, autophagy-related factors play important roles in psoriasis (Wu & Adamopoulos, 2017). Our study identified that the ATG5 gene had crucial roles in the pathogenesis and might be a therapeutic target in psoriasis. Also, ATG5 might be a paeonol-targeted gene.

Aberrant cell growth, differentiation, and inflammation are the major physiologic characteristics of psoriasis lesions (Victor & Gottlieb, 2002; Wang et al., 2020; Zhang et al., 2015). Autophagy in keratinocytes correlates with disease severity in psoriasis patients (Wang et al., 2020). Wang et al., (2020) showed that the high mobility group box 1 (HMGB1)-associated autosecretion is effective in regulating cutaneous inflammation in autophagy-efficient (*ATG5*^*f*∕*f*^) keratinocytes. Another study by Peng et al. (2019) showed that the knockout of *ATG5* in proximal tubular epithelial cells impaired inflammation through activating the NF-*κ*B pathway. Our study demonstrated that a cluster of psoriasis- and immunity-related genes, including the ATG5 gene, were upregulated in psoriatic lesions. These results were in line with the fact that the activation of inflammation and autophagy are the major physiologic features of psoriasis lesions.

We found that the *ATG5* gene had a distinctive role in psoriasis. It is a target of paeonol which has anti-inflammatory (Lou et al., 2017; Zhai et al., 2017) and anti-psoriasis effects (Meng et al., 2017). Zhai et al. (2017) reported that paeonol suppressed the production of TNF-a, IL-6, and IL-1*β* in rheumatoid arthritis fibroblast-like synoviocytes by suppressing NF-*κ*B. Paeonol suppressed IL-1*β*-induced inflammation through several pathways, including the PI3K/Akt/NF-*κ*B pathway (Jin et al., 2016), MAPK/ERK/p38 signaling pathway (Lou et al., 2017), mTOR pathway (Cao et al., 2016; Dai, Zhou & Song, 2018), and STAT3 signaling (Zhao et al., 2015). Also, paeonol blocks the Akt/mTOR pathway and induces cytoprotective autophagy in ovarian cancer cells (Gao et al., 2019a). Our study showed that the *ATG5* gene interacted with the factors including mTOR, Akt1, IL-6, and TNF, and all of them are the potential therapeutic targets of paeonol. Victor & Gottlieb (2002) showed that TNF-*α* overexpression triggered the apoptotic progression and initiated the development of psoriatic lesions. Our in vitro experiments showed that paeonol suppressed IL-22/TNF- *α*-induced proliferation, ATG5 expression, and inflammation in HaCaT cells. These findings showed that the *ATG5* gene might have a crucial role in psoriasis onset and might be a target for psoriasis management.

ATG5-mediated autophagy is crucial for a wide range of biological processes, including DNA damage, cell proliferation, apoptosis, inflammation, differentiation, and drug resistance (Han et al., 2020; Kim et al., 2020b; Peng et al., 2019; Yue et al., 2019). The inhibition of autophagy might induce an increase in cell apoptosis and inflammation and a reduction in cell proliferation in hepatocytes (Wu & Adamopoulos, 2017; Zhang et al., 2020). Also, autophagy inhibition results in exacerbating skin inflammation and increased disease severity (Wu & Adamopoulos, 2017). Zhang et al. (2020) revealed that acetaminophen-induced inflammation, apoptosis, and proliferation inhibition in the hepatocyte L-02 cell line could be enhanced by inhibiting autophagy and rescued by activating autophagy, respectively. They showed that the inhibition of autophagy in hepatocytes increased inflammation cytokines including IL-18 and IL-1*β*. However, Yue et al. (2019) and Feng et al. (2019) showed that the induction of autophagy ameliorates in vitro psoriasis. Our research showed that the *ATG5* gene was upregulated in the in vitro cellular psoriatic model. The inhibition of *ATG5* showed a supportive effect on paeonol-induced cell proliferation inhibition, Beclin 1 reduction, and cell apoptosis in HaCaT cells. The increased IL-6 and IL-1*β* contents by si-ATG5 transfection showed that autophagy inhibition enhanced inflammation in IL-22/TNF-*α*-treated HaCaT cells. Also, the decreased IL-6 and IL-1*β* contents by pc-ATG5 transfection showed that ATG5-mediated autophagy might be crucial for preventing psoriasis. These findings suggested that the *ATG5* gene might have an important role in psoriasis.

Autophagy deficiency in keratinocytes increased the production of inflammatory cytokines (Lee et al., 2011). However, previous studies showed that the mixture of five proinflammatory cytokines (IL-17A, IL-22, Oncostatin-M, TNF- *α*, and IL-1 *α*) (Kim et al., 2020a), IL-17A (Varshney & Saini, 2018), and TNF-*α* (Yue et al., 2019) treatments significantly decreased autophagy and *ATG5* expression in HaCaT cells. However, our present study showed that the ATG5 gene was upregulated in HaCaT cells stimulated by IL-22/TNF-*α* cytokines. Also, it was increased in the lesional skins compared with non-lesional skins from patients with psoriasis. This difference might due to the distinct stimulation strategies (Kim et al., 2020a; Varshney & Saini, 2018; Yue et al., 2019). Besides, our present study showed that paeonol was able to reduce the expression levels of ATG5, IL-6, and IL-1*β* upon IL-22/TNF-*α* treatment. However, prior inhibition or overexpression of ATG5 blocked the influence of IL-22/TNF- *α* treatment on cell inflammation, proliferation, and apoptosis. These findings indicated that the effect of paeonol on HaCaT cells was ATG5-dependent. However, the exact mechanism of autophagy in psoriasis should be further validated.

## Conclusions

In conclusion, we confirmed that *ATG5*-dependent autophagy was of great value in psoriasis. *ATG5* was increased in lesional skin tissues compared with non-lesional tissues. The inhibition of *ATG5* promoted inflammation in HaCaT cells and the overexpression of *ATG5* prevented IL-22/TNF- *α*-induced inflammation in HaCaT cells. Also, the effect of paeonol on in vitro psoriatic model was ATG5-dependent. The inhibition of *ATG5* might be a targeting management strategy for in vitro psoriasis. However, more experiments should be performed to validate the association of autophagy with psoriasis and the probability of targeting autophagy for the management of psoriasis.

##  Supplemental Information

10.7717/peerj.11278/supp-1 Supplemental Information 1 The correlation of the differentially expressed genes between the four expression datasets (A), the correlation analysis of gene expression level in the training and validation datasets. (B) the correlation of node connection in the training and validation datasets.

10.7717/peerj.11278/supp-2 Supplemental Information 2 The protein-protein interaction (PPI) network of the differentially expressed genes in the two positive modules The node colors denote the degree of significance and the closer to the red the node gene will has a higher degree of expression.

10.7717/peerj.11278/supp-3 Supplemental Information 3 The list of the 779 differentially expressed genes between the lesion and non-lesion samples across the four datasets

10.7717/peerj.11278/supp-4 Supplemental Information 4 The functional enrichment analysis of differentially expressed genes in the protein–protein interaction network

10.7717/peerj.11278/supp-5 Supplemental Information 5 The list of the 133 upregulated genes including in the protein–protein interaction network

10.7717/peerj.11278/supp-6 Supplemental Information 6 Raw data: Figs. 6, 7, 8 and 9

10.7717/peerj.11278/supp-7 Supplemental Information 7 Raw protein bands Protein bands of ATG5, Beclin 1, and p62.

## Additional Information and Declarations

### Data Availability

The following information was supplied regarding data availability: The original microarray data is available at NCBI GEO: GSE30999, GSE13355, GSE14905, and GSE41662. All data generated or analyzed during this study are included in this published article.
