# Supplementary figures and images for: The paeonol target gene autophagy-related 5 has a potential therapeutic value in psoriasis treatment
Source: PeerJ. 2021 May 25;9:e11278. doi: 10.7717/peerj.11278 (PMC8162242; doi:10.7717/peerj.11278)

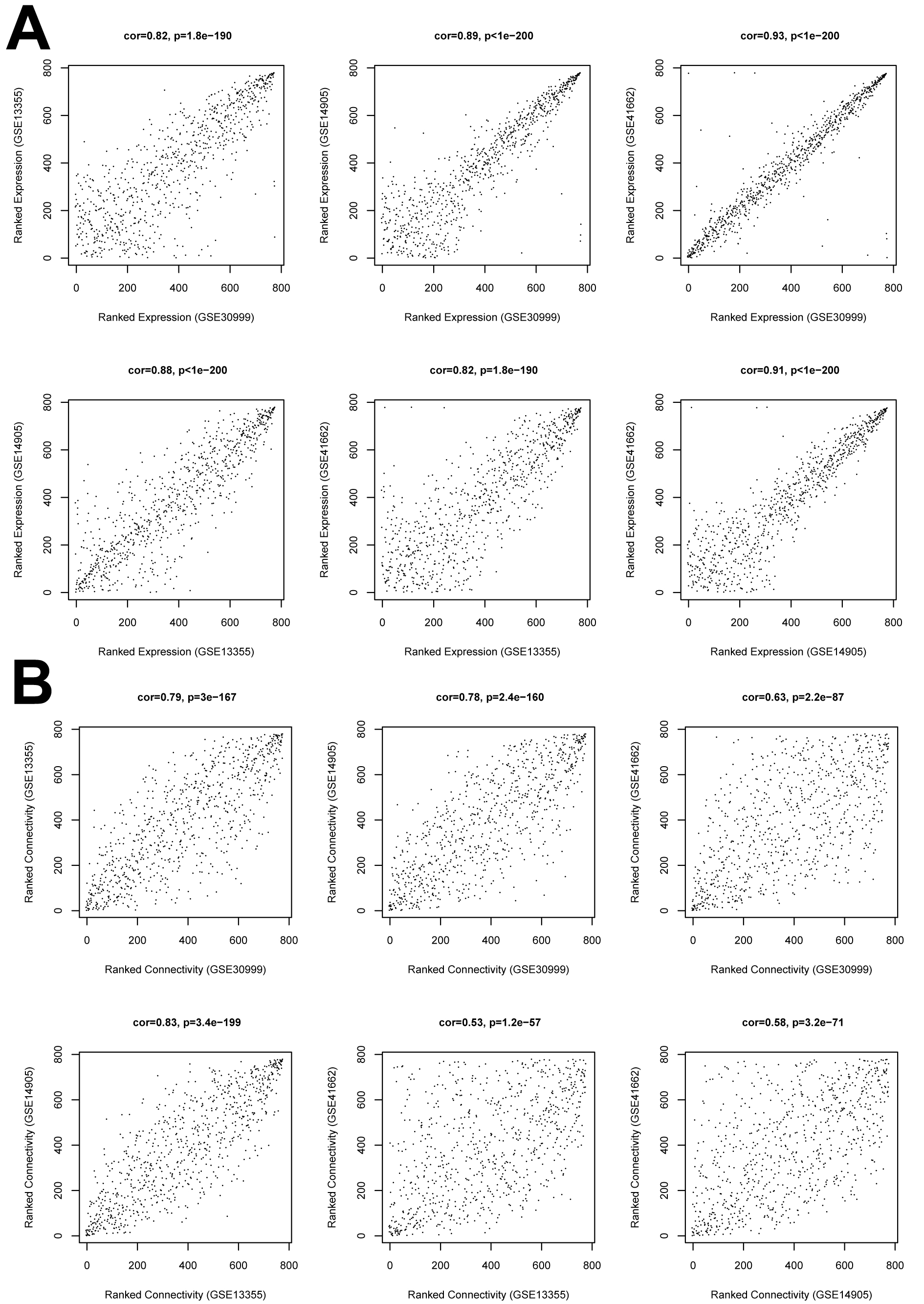

Supplement: Supplemental Information 1 — (A), the correlation analysis of gene expression level in the training and validation datasets. (B) the correlation of node connection in the training and validation datasets. [file peerj-09-11278-s001.jpg]

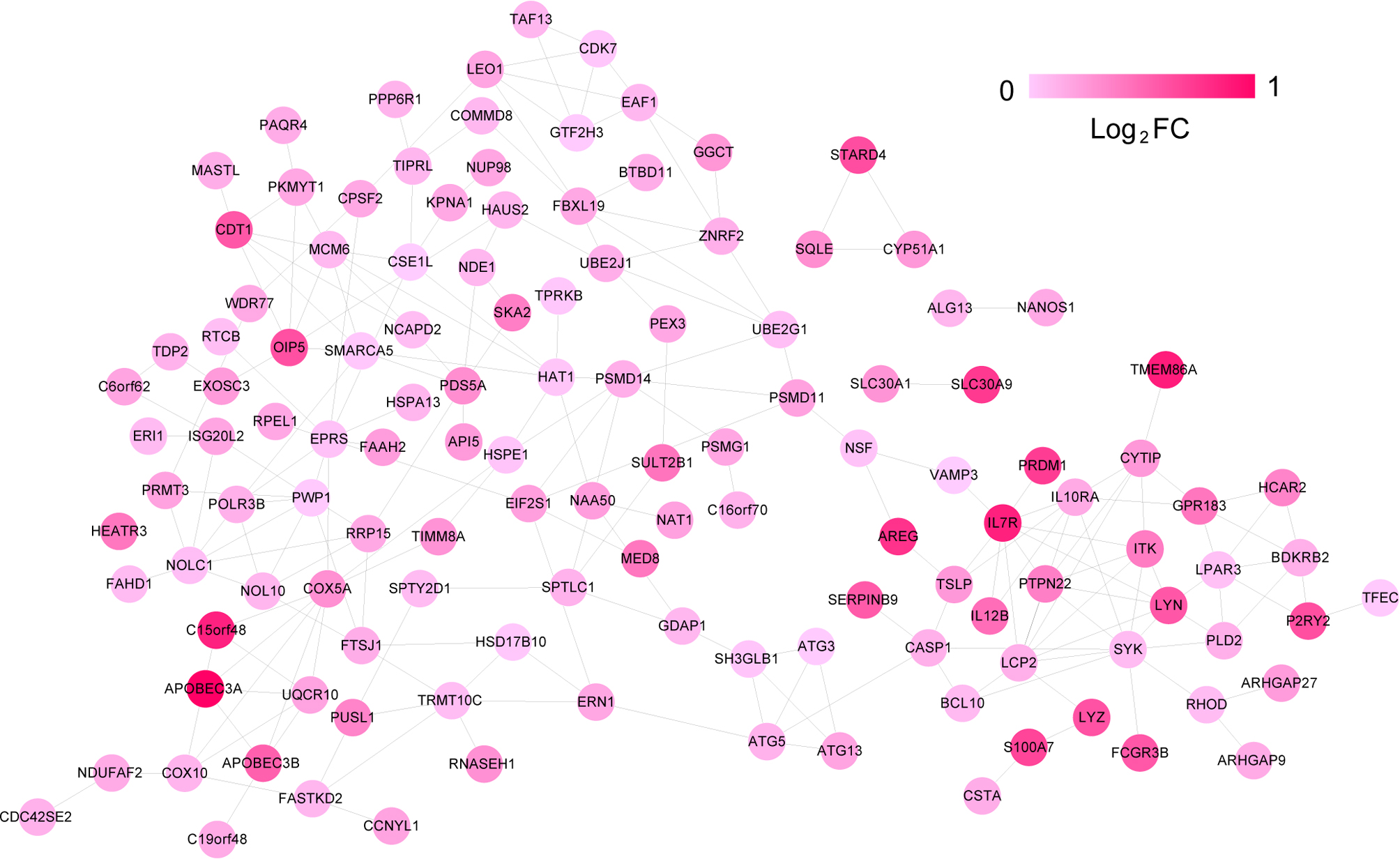

Supplement: Supplemental Information 2 — The node colors denote the degree of significance and the closer to the red the node gene will has a higher degree of expression. [file peerj-09-11278-s002.jpg]
