# Supplementary material for: The paeonol target gene autophagy-related 5 has a potential therapeutic value in psoriasis treatment
Source: PeerJ. 2021 May 25;9:e11278. doi: 10.7717/peerj.11278 (PMC8162242; doi:10.7717/peerj.11278)
Supplement: Supplemental Information 3 [file peerj-09-11278-s003.doc]

Table S1. The list of the 779 differentially expressed genes between the lesion and non-lesion samples across the four datasets.

| Gene | p | FDR | Q | Qp | tau2 |  | logFC |  |  |
| --- | --- | --- | --- | --- | --- | --- | --- | --- | --- |
|  |  |  |  |  |  | GSE30999 | GSE13355 | GSE14905 | GSE41662 |
| SCGB1D2 | 1.00E-20 | 4.53E-20 | 1.200 | 0.753 | 0.000 | -0.493 | -0.622 | -0.692 | -1.410 |
| FADS1 | 1.00E-20 | 4.53E-20 | 1.232 | 0.745 | 0.000 | -0.654 | -0.567 | -0.637 | -1.340 |
| KRT79 | 1.00E-20 | 4.53E-20 | 0.265 | 0.966 | 0.000 | -0.680 | -0.572 | -0.687 | -1.320 |
| PM20D1 | 1.00E-20 | 4.53E-20 | 0.137 | 0.987 | 0.000 | -0.962 | -0.848 | -1.151 | -1.313 |
| CRAT | 1.00E-20 | 4.53E-20 | 1.706 | 0.636 | 0.000 | -0.632 | -0.552 | -0.494 | -1.236 |
| CLDN8 | 1.00E-20 | 4.53E-20 | 2.756 | 0.431 | 0.000 | -0.733 | -0.715 | -1.033 | -1.234 |
| HSPB6 | 1.00E-20 | 4.53E-20 | 1.879 | 0.598 | 0.000 | -0.455 | -0.363 | -0.469 | -1.206 |
| ADIPOQ | 1.00E-20 | 4.53E-20 | 1.815 | 0.612 | 0.000 | -0.562 | -0.583 | -0.565 | -1.204 |
| LPL | 1.00E-20 | 4.53E-20 | 2.845 | 0.416 | 0.000 | -0.540 | -0.554 | -0.588 | -1.115 |
| GAL | 1.00E-20 | 4.53E-20 | 0.476 | 0.924 | 0.000 | -1.269 | -0.829 | -1.006 | -1.094 |
| APOC1 | 1.00E-20 | 4.53E-20 | 1.880 | 0.598 | 0.000 | -0.693 | -0.388 | -0.426 | -1.087 |
| MGST1 | 1.00E-20 | 4.53E-20 | 2.968 | 0.397 | 0.000 | -0.575 | -0.508 | -0.487 | -1.074 |
| SEMA3G | 1.00E-20 | 4.53E-20 | 1.880 | 0.598 | 0.000 | -0.458 | -0.465 | -0.645 | -1.072 |
| SORBS1 | 1.00E-20 | 4.53E-20 | 1.861 | 0.602 | 0.000 | -0.395 | -0.529 | -0.683 | -1.065 |
| WFDC3 | 1.00E-20 | 4.53E-20 | 0.211 | 0.976 | 0.000 | -0.969 | -0.495 | -0.767 | -1.065 |
| MUC1 | 1.00E-20 | 4.53E-20 | 2.448 | 0.485 | 0.000 | -0.507 | -0.438 | -0.453 | -1.059 |
| ZDHHC11 | 1.00E-20 | 4.53E-20 | 0.569 | 0.903 | 0.000 | -0.686 | -0.520 | -0.740 | -1.045 |
| FABP4 | 1.00E-20 | 4.53E-20 | 2.476 | 0.480 | 0.000 | -0.781 | -0.507 | -0.661 | -0.952 |
| MLPH | 1.00E-20 | 4.53E-20 | 1.461 | 0.691 | 0.000 | -0.395 | -0.372 | -0.466 | -0.928 |
| CYP4F8 | 1.00E-20 | 4.53E-20 | 1.578 | 0.664 | 0.000 | -0.951 | -0.391 | -0.695 | -0.915 |
| LEP | 1.00E-20 | 4.53E-20 | 2.863 | 0.413 | 0.000 | -0.872 | -0.610 | -0.584 | -0.910 |
| EPCAM | 1.00E-20 | 4.53E-20 | 0.244 | 0.970 | 0.000 | -0.577 | -0.516 | -0.573 | -0.909 |
| HAO2 | 1.00E-20 | 4.53E-20 | 2.730 | 0.435 | 0.000 | -1.444 | -0.490 | -0.797 | -0.905 |
| KIT | 1.00E-20 | 4.53E-20 | 0.736 | 0.865 | 0.000 | -0.387 | -0.436 | -0.546 | -0.900 |
| TAGLN | 1.00E-20 | 4.53E-20 | 2.926 | 0.403 | 0.000 | -0.615 | -0.414 | -0.455 | -0.898 |
| RETREG1 | 1.00E-20 | 4.53E-20 | 2.692 | 0.442 | 0.000 | -0.425 | -0.514 | -0.572 | -0.896 |
| EN1 | 1.00E-20 | 4.53E-20 | 2.765 | 0.429 | 0.000 | -0.444 | -0.420 | -0.508 | -0.896 |
| AHNAK2 | 1.00E-20 | 4.53E-20 | 1.599 | 0.660 | 0.000 | -0.510 | -0.518 | -1.027 | -0.894 |
| ELOVL3 | 1.00E-20 | 4.53E-20 | 1.593 | 0.661 | 0.000 | -1.402 | -0.812 | -1.083 | -0.893 |
| DEPP1 | 1.00E-20 | 4.53E-20 | 2.142 | 0.544 | 0.000 | -0.602 | -0.420 | -0.473 | -0.887 |
| GPAM | 1.00E-20 | 4.53E-20 | 0.470 | 0.926 | 0.000 | -0.466 | -0.473 | -0.470 | -0.887 |
| GRB14 | 1.00E-20 | 4.53E-20 | 1.709 | 0.635 | 0.000 | -0.568 | -0.356 | -0.574 | -0.884 |
| POU6F1 | 1.00E-20 | 4.53E-20 | 0.796 | 0.850 | 0.000 | -0.578 | -0.369 | -0.629 | -0.877 |
| C11orf96 | 1.00E-20 | 4.53E-20 | 1.156 | 0.763 | 0.000 | -0.330 | -0.369 | -0.371 | -0.874 |
| CES1 | 1.00E-20 | 4.53E-20 | 2.371 | 0.499 | 0.000 | -0.651 | -0.565 | -0.579 | -0.868 |
| PELI2 | 1.00E-20 | 4.53E-20 | 2.062 | 0.560 | 0.000 | -0.468 | -0.475 | -0.657 | -0.867 |
| ITM2A | 1.00E-20 | 4.53E-20 | 2.002 | 0.572 | 0.000 | -0.648 | -0.558 | -0.523 | -0.851 |
| PDZK1 | 1.00E-20 | 4.53E-20 | 0.413 | 0.938 | 0.000 | -0.631 | -0.436 | -0.763 | -0.846 |
| GLDC | 1.00E-20 | 4.53E-20 | 1.797 | 0.616 | 0.000 | -1.210 | -0.492 | -0.650 | -0.837 |
| HSD11B2 | 1.00E-20 | 4.53E-20 | 2.186 | 0.535 | 0.000 | -0.486 | -0.414 | -0.515 | -0.830 |
| PMEPA1 | 1.00E-20 | 4.53E-20 | 2.334 | 0.506 | 0.000 | -0.346 | -0.369 | -0.397 | -0.828 |
| IGFBP6 | 1.00E-20 | 4.53E-20 | 1.895 | 0.594 | 0.000 | -0.610 | -0.503 | -0.500 | -0.828 |
| CYBRD1 | 1.00E-20 | 4.53E-20 | 0.336 | 0.953 | 0.000 | -0.370 | -0.416 | -0.470 | -0.826 |
| OLFML2A | 1.00E-20 | 4.53E-20 | 1.942 | 0.585 | 0.000 | -0.423 | -0.436 | -0.512 | -0.824 |
| PRKAR2B | 1.00E-20 | 4.53E-20 | 1.295 | 0.730 | 0.000 | -0.452 | -0.377 | -0.446 | -0.823 |
| CACNA2D1 | 1.00E-20 | 4.53E-20 | 0.808 | 0.847 | 0.000 | -0.494 | -0.515 | -0.571 | -0.822 |
| CAVIN2 | 1.00E-20 | 4.53E-20 | 1.812 | 0.612 | 0.000 | -0.434 | -0.457 | -0.522 | -0.819 |
| ANKRD36C | 1.00E-20 | 4.53E-20 | 2.153 | 0.541 | 0.000 | -0.392 | -0.460 | -0.580 | -0.817 |
| FNBP1L | 1.00E-20 | 4.53E-20 | 2.672 | 0.445 | 0.000 | -0.396 | -0.426 | -0.482 | -0.808 |
| GPC6 | 1.00E-20 | 4.53E-20 | 2.590 | 0.459 | 0.000 | -0.805 | -0.439 | -0.619 | -0.802 |
| EFEMP1 | 1.00E-20 | 4.53E-20 | 0.148 | 0.986 | 0.000 | -0.417 | -0.485 | -0.508 | -0.799 |
| GPD1L | 1.00E-20 | 4.53E-20 | 2.237 | 0.525 | 0.000 | -0.386 | -0.418 | -0.531 | -0.793 |
| TBC1D24 | 1.00E-20 | 4.53E-20 | 0.883 | 0.830 | 0.000 | -0.421 | -0.414 | -0.528 | -0.789 |
| NR3C2 | 1.00E-20 | 4.53E-20 | 1.808 | 0.613 | 0.000 | -0.824 | -0.615 | -0.742 | -0.786 |
| TGFBR2 | 1.00E-20 | 4.53E-20 | 0.258 | 0.968 | 0.000 | -0.336 | -0.358 | -0.438 | -0.781 |
| GHR | 1.00E-20 | 4.53E-20 | 1.632 | 0.652 | 0.000 | -0.372 | -0.428 | -0.409 | -0.763 |
| SOAT1 | 1.00E-20 | 4.53E-20 | 1.042 | 0.791 | 0.000 | -0.456 | -0.399 | -0.495 | -0.759 |
| PDCD4 | 1.00E-20 | 4.53E-20 | 1.240 | 0.743 | 0.000 | -0.349 | -0.450 | -0.469 | -0.755 |
| BPY2 | 1.00E-20 | 4.53E-20 | 1.538 | 0.673 | 0.000 | -1.299 | -0.448 | -0.649 | -0.748 |
| EMCN | 1.00E-20 | 4.53E-20 | 2.993 | 0.393 | 0.000 | -0.354 | -0.406 | -0.438 | -0.745 |
| PTPRM | 1.00E-20 | 4.53E-20 | 0.116 | 0.990 | 0.000 | -0.350 | -0.354 | -0.385 | -0.737 |
| TMEM178A | 1.00E-20 | 4.53E-20 | 0.541 | 0.910 | 0.000 | -0.666 | -0.418 | -0.613 | -0.737 |
| MAL | 1.00E-20 | 4.53E-20 | 2.098 | 0.552 | 0.000 | -0.584 | -0.393 | -0.409 | -0.731 |
| AKAP12 | 1.00E-20 | 4.53E-20 | 0.343 | 0.952 | 0.000 | -0.438 | -0.393 | -0.509 | -0.723 |
| PPP1R14A | 1.00E-20 | 4.53E-20 | 1.085 | 0.781 | 0.000 | -0.631 | -0.395 | -0.687 | -0.718 |
| AACS | 1.00E-20 | 4.53E-20 | 0.463 | 0.927 | 0.000 | -0.363 | -0.397 | -0.408 | -0.717 |
| THRSP | 1.00E-20 | 4.53E-20 | 2.429 | 0.488 | 0.000 | -1.271 | -0.680 | -0.790 | -0.715 |
| INSIG1 | 1.00E-20 | 4.53E-20 | 1.490 | 0.685 | 0.000 | -0.518 | -0.389 | -0.419 | -0.715 |
| ACSS2 | 1.00E-20 | 4.53E-20 | 2.560 | 0.465 | 0.000 | -0.360 | -0.357 | -0.346 | -0.715 |
| NOSTRIN | 1.00E-20 | 4.53E-20 | 2.461 | 0.482 | 0.000 | -0.417 | -0.408 | -0.542 | -0.712 |
| HSPG2 | 1.00E-20 | 4.53E-20 | 2.888 | 0.409 | 0.000 | -0.348 | -0.367 | -0.388 | -0.712 |
| KLF2 | 1.00E-20 | 4.53E-20 | 1.350 | 0.717 | 0.000 | -0.420 | -0.412 | -0.395 | -0.709 |
| CIRBP | 1.00E-20 | 4.53E-20 | 1.619 | 0.655 | 0.000 | -0.436 | -0.433 | -0.874 | -0.704 |
| RNF128 | 1.00E-20 | 4.53E-20 | 1.149 | 0.765 | 0.000 | -0.490 | -0.501 | -0.430 | -0.696 |
| KRT4 | 1.00E-20 | 4.53E-20 | 2.001 | 0.572 | 0.000 | -1.188 | -0.311 | -0.373 | -0.694 |
| PCM1 | 1.00E-20 | 4.53E-20 | 1.480 | 0.687 | 0.000 | -0.379 | -0.469 | -0.499 | -0.693 |
| TSPYL2 | 1.00E-20 | 4.53E-20 | 1.071 | 0.784 | 0.000 | -0.390 | -0.354 | -0.488 | -0.688 |
| MTERF2 | 1.00E-20 | 4.53E-20 | 0.518 | 0.915 | 0.000 | -0.364 | -0.378 | -0.451 | -0.688 |
| NSUN6 | 1.00E-20 | 4.53E-20 | 0.345 | 0.951 | 0.000 | -0.371 | -0.337 | -0.490 | -0.687 |
| MFSD2A | 1.00E-20 | 4.53E-20 | 2.445 | 0.485 | 0.000 | -0.361 | -0.396 | -0.479 | -0.686 |
| CBR4 | 1.00E-20 | 4.53E-20 | 2.111 | 0.550 | 0.000 | -0.423 | -0.376 | -0.395 | -0.685 |
| CTIF | 1.00E-20 | 4.53E-20 | 2.022 | 0.568 | 0.000 | -0.406 | -0.344 | -0.387 | -0.685 |
| LUC7L3 | 1.00E-20 | 4.53E-20 | 1.660 | 0.646 | 0.000 | -0.336 | -0.389 | -0.441 | -0.684 |
| LAMB2 | 1.00E-20 | 4.53E-20 | 2.389 | 0.496 | 0.000 | -0.357 | -0.364 | -0.407 | -0.682 |
| ACSL1 | 1.00E-20 | 4.53E-20 | 0.442 | 0.931 | 0.000 | -0.327 | -0.392 | -0.367 | -0.682 |
| ACSF2 | 1.00E-20 | 4.53E-20 | 1.786 | 0.618 | 0.000 | -0.514 | -0.368 | -0.469 | -0.682 |
| ACOX2 | 1.00E-20 | 4.53E-20 | 0.348 | 0.951 | 0.000 | -0.773 | -0.429 | -0.620 | -0.681 |
| EFNA5 | 1.00E-20 | 4.53E-20 | 0.926 | 0.819 | 0.000 | -0.375 | -0.439 | -0.423 | -0.678 |
| MUSTN1 | 1.00E-20 | 4.53E-20 | 1.066 | 0.785 | 0.000 | -0.404 | -0.396 | -0.479 | -0.677 |
| TIMP2 | 1.00E-20 | 4.53E-20 | 2.062 | 0.560 | 0.000 | -0.313 | -0.376 | -0.393 | -0.676 |
| PECR | 1.00E-20 | 4.53E-20 | 2.550 | 0.466 | 0.000 | -0.393 | -0.376 | -0.351 | -0.675 |
| ZNF273 | 1.00E-20 | 4.53E-20 | 0.897 | 0.826 | 0.000 | -0.417 | -0.381 | -0.663 | -0.674 |
| MAGED2 | 1.00E-20 | 4.53E-20 | 2.990 | 0.393 | 0.000 | -0.319 | -0.337 | -0.346 | -0.663 |
| ADGRL4 | 1.00E-20 | 4.53E-20 | 1.089 | 0.780 | 0.000 | -0.347 | -0.347 | -0.372 | -0.661 |
| NOL3 | 1.00E-20 | 4.53E-20 | 2.722 | 0.436 | 0.000 | -0.305 | -0.296 | -0.362 | -0.660 |
| RUFY3 | 1.00E-20 | 4.53E-20 | 0.974 | 0.808 | 0.000 | -0.358 | -0.456 | -0.401 | -0.652 |
| CFH | 1.00E-20 | 4.53E-20 | 1.246 | 0.742 | 0.000 | -0.337 | -0.343 | -0.389 | -0.651 |
| TWIST1 | 1.00E-20 | 4.53E-20 | 2.128 | 0.546 | 0.000 | -0.310 | -0.379 | -0.372 | -0.649 |
| HOMER2 | 1.00E-20 | 4.53E-20 | 0.539 | 0.910 | 0.000 | -0.470 | -0.382 | -0.467 | -0.642 |
| SIX1 | 1.00E-20 | 4.53E-20 | 2.828 | 0.419 | 0.000 | -0.718 | -0.300 | -0.640 | -0.637 |
| PRR15L | 1.00E-20 | 4.53E-20 | 2.163 | 0.539 | 0.000 | -1.044 | -0.464 | -0.675 | -0.633 |
| HBA1 | 1.00E-20 | 4.53E-20 | 1.946 | 0.584 | 0.000 | -0.583 | -0.403 | -0.413 | -0.633 |
| GRK3 | 1.00E-20 | 4.53E-20 | 2.950 | 0.399 | 0.000 | -0.376 | -0.415 | -0.439 | -0.632 |
| FAM221A | 1.00E-20 | 4.53E-20 | 2.593 | 0.459 | 0.000 | -0.486 | -0.407 | -0.641 | -0.630 |
| TCEAL3 | 1.00E-20 | 4.53E-20 | 2.301 | 0.512 | 0.000 | -0.507 | -0.416 | -0.463 | -0.629 |
| ST6GALNAC2 | 1.00E-20 | 4.53E-20 | 1.642 | 0.650 | 0.000 | -0.309 | -0.372 | -0.390 | -0.626 |
| SERHL2 | 1.00E-20 | 4.53E-20 | 1.146 | 0.766 | 0.000 | -0.641 | -0.408 | -0.822 | -0.626 |
| SLC29A1 | 1.00E-20 | 4.53E-20 | 2.556 | 0.465 | 0.000 | -0.408 | -0.339 | -0.479 | -0.626 |
| COL5A3 | 1.00E-20 | 4.53E-20 | 2.948 | 0.400 | 0.000 | -0.321 | -0.284 | -0.340 | -0.625 |
| ARGLU1 | 1.00E-20 | 4.53E-20 | 2.606 | 0.456 | 0.000 | -0.416 | -0.383 | -0.559 | -0.624 |
| SESTD1 | 1.00E-20 | 4.53E-20 | 2.182 | 0.536 | 0.000 | -0.514 | -0.458 | -0.504 | -0.619 |
| CCDC107 | 1.00E-20 | 4.53E-20 | 1.321 | 0.724 | 0.000 | -0.400 | -0.328 | -0.350 | -0.618 |
| EID1 | 1.00E-20 | 4.53E-20 | 2.869 | 0.412 | 0.000 | -0.320 | -0.365 | -0.337 | -0.618 |
| VPS13C | 1.00E-20 | 4.53E-20 | 2.939 | 0.401 | 0.000 | -0.443 | -0.442 | -0.446 | -0.616 |
| ILK | 1.00E-20 | 4.53E-20 | 1.838 | 0.607 | 0.000 | -0.328 | -0.318 | -0.342 | -0.616 |
| KRT31 | 1.00E-20 | 4.53E-20 | 1.905 | 0.592 | 0.000 | -0.422 | -0.488 | -0.569 | -0.616 |
| PDGFRB | 1.00E-20 | 4.53E-20 | 2.972 | 0.396 | 0.000 | -0.355 | -0.335 | -0.334 | -0.613 |
| CD81 | 1.00E-20 | 4.53E-20 | 2.261 | 0.520 | 0.000 | -0.311 | -0.342 | -0.363 | -0.610 |
| CXCL12 | 1.00E-20 | 4.53E-20 | 1.503 | 0.682 | 0.000 | -0.314 | -0.307 | -0.325 | -0.609 |
| GLG1 | 1.00E-20 | 4.53E-20 | 2.926 | 0.403 | 0.000 | -0.347 | -0.342 | -0.421 | -0.607 |
| ZNF284 | 1.00E-20 | 4.53E-20 | 0.841 | 0.840 | 0.000 | -0.379 | -0.337 | -0.431 | -0.607 |
| RASA3 | 1.00E-20 | 4.53E-20 | 1.744 | 0.627 | 0.000 | -0.414 | -0.318 | -0.398 | -0.604 |
| VGLL3 | 1.00E-20 | 4.53E-20 | 2.614 | 0.455 | 0.000 | -0.382 | -0.408 | -0.415 | -0.603 |
| LATS2 | 1.00E-20 | 4.53E-20 | 1.307 | 0.727 | 0.000 | -0.311 | -0.335 | -0.364 | -0.602 |
| PNISR | 1.00E-20 | 4.53E-20 | 0.682 | 0.877 | 0.000 | -0.431 | -0.374 | -0.358 | -0.602 |
| ZNF880 | 1.00E-20 | 4.53E-20 | 2.320 | 0.509 | 0.000 | -0.528 | -0.371 | -0.379 | -0.601 |
| GPIHBP1 | 1.00E-20 | 4.53E-20 | 1.435 | 0.697 | 0.000 | -0.860 | -0.347 | -0.622 | -0.599 |
| MMRN2 | 1.00E-20 | 4.53E-20 | 1.297 | 0.730 | 0.000 | -0.354 | -0.386 | -0.349 | -0.597 |
| SENP7 | 1.00E-20 | 4.53E-20 | 2.953 | 0.399 | 0.000 | -0.346 | -0.351 | -0.394 | -0.595 |
| ORAI3 | 1.00E-20 | 4.53E-20 | 1.173 | 0.760 | 0.000 | -0.349 | -0.355 | -0.404 | -0.594 |
| SEC62 | 1.00E-20 | 4.53E-20 | 2.593 | 0.459 | 0.000 | -0.449 | -0.452 | -0.463 | -0.594 |
| AGPAT3 | 2.88E-06 | 1.09E-05 | 2.248 | 0.523 | 0.000 | -0.306 | -0.284 | -0.311 | -0.594 |
| FREM2 | 1.00E-20 | 4.53E-20 | 2.462 | 0.482 | 0.000 | -0.940 | -0.337 | -0.507 | -0.590 |
| ITGA7 | 1.00E-20 | 4.53E-20 | 2.492 | 0.477 | 0.000 | -0.554 | -0.375 | -0.459 | -0.589 |
| DTX4 | 1.00E-20 | 4.53E-20 | 1.569 | 0.666 | 0.000 | -0.599 | -0.338 | -0.371 | -0.588 |
| TXNL1 | 1.00E-20 | 4.53E-20 | 1.951 | 0.583 | 0.000 | -0.608 | -0.452 | -0.616 | -0.587 |
| SLC25A27 | 1.00E-20 | 4.53E-20 | 1.883 | 0.597 | 0.000 | -0.405 | -0.328 | -0.639 | -0.586 |
| SF3B1 | 1.00E-20 | 4.53E-20 | 2.772 | 0.428 | 0.000 | -0.378 | -0.357 | -0.456 | -0.584 |
| GNPAT | 1.00E-20 | 4.53E-20 | 1.778 | 0.620 | 0.000 | -0.333 | -0.387 | -0.355 | -0.584 |
| SRSF11 | 1.00E-20 | 4.53E-20 | 2.471 | 0.481 | 0.000 | -0.333 | -0.445 | -0.431 | -0.583 |
| KATNAL1 | 1.00E-20 | 4.53E-20 | 0.684 | 0.877 | 0.000 | -0.380 | -0.425 | -0.438 | -0.582 |
| TNS4 | 1.00E-20 | 4.53E-20 | 2.635 | 0.451 | 0.000 | -0.294 | -0.309 | -0.403 | -0.582 |
| PLIN2 | 1.00E-20 | 4.53E-20 | 0.129 | 0.988 | 0.000 | -0.341 | -0.376 | -0.335 | -0.581 |
| CLEC14A | 1.00E-20 | 4.53E-20 | 2.608 | 0.456 | 0.000 | -0.389 | -0.320 | -0.378 | -0.580 |
| AQP7 | 1.00E-20 | 4.53E-20 | 1.412 | 0.703 | 0.000 | -0.822 | -0.325 | -0.319 | -0.580 |
| MEF2D | 1.00E-20 | 4.53E-20 | 2.254 | 0.521 | 0.000 | -0.401 | -0.384 | -0.402 | -0.580 |
| PPARG | 1.00E-20 | 4.53E-20 | 2.798 | 0.424 | 0.000 | -0.979 | -0.378 | -0.583 | -0.579 |
| CCDC85A | 1.00E-20 | 4.53E-20 | 0.760 | 0.859 | 0.000 | -0.558 | -0.349 | -0.490 | -0.578 |
| ZBED3 | 1.00E-20 | 4.53E-20 | 1.080 | 0.782 | 0.000 | -0.444 | -0.352 | -0.519 | -0.573 |
| UBN2 | 1.00E-20 | 4.53E-20 | 2.221 | 0.528 | 0.000 | -0.531 | -0.475 | -0.716 | -0.572 |
| CCNI | 1.00E-20 | 4.53E-20 | 1.338 | 0.720 | 0.000 | -0.309 | -0.349 | -0.325 | -0.569 |
| ZBTB4 | 1.00E-20 | 4.53E-20 | 1.944 | 0.584 | 0.000 | -0.365 | -0.367 | -0.404 | -0.569 |
| ESRRG | 1.00E-20 | 4.53E-20 | 2.124 | 0.547 | 0.000 | -0.767 | -0.384 | -0.484 | -0.568 |
| PNMA8A | 1.15E-06 | 4.51E-06 | 2.990 | 0.393 | 0.000 | -0.575 | -0.397 | -0.403 | -0.567 |
| PPP1R1A | 1.00E-20 | 4.53E-20 | 0.584 | 0.900 | 0.000 | -0.897 | -0.322 | -0.490 | -0.566 |
| ENC1 | 1.00E-20 | 4.53E-20 | 0.961 | 0.811 | 0.000 | -0.409 | -0.324 | -0.359 | -0.564 |
| NEMF | 1.00E-20 | 4.53E-20 | 2.135 | 0.545 | 0.000 | -0.312 | -0.330 | -0.337 | -0.562 |
| IGFBP7 | 1.00E-20 | 4.53E-20 | 2.450 | 0.484 | 0.000 | -0.296 | -0.303 | -0.305 | -0.562 |
| WEE1 | 1.00E-20 | 4.53E-20 | 2.248 | 0.522 | 0.000 | -0.621 | -0.438 | -0.829 | -0.562 |
| TTC14 | 1.00E-20 | 4.53E-20 | 2.532 | 0.469 | 0.000 | -0.320 | -0.354 | -0.407 | -0.561 |
| DCXR | 0.009525 | 0.019671 | 1.805 | 0.614 | 0.000 | -0.278 | -0.269 | -0.296 | -0.560 |
| PCGF2 | 1.00E-20 | 4.53E-20 | 2.829 | 0.419 | 0.000 | -0.326 | -0.364 | -0.374 | -0.560 |
| NRP1 | 1.00E-20 | 4.53E-20 | 1.116 | 0.773 | 0.000 | -0.345 | -0.322 | -0.375 | -0.560 |
| IVD | 1.00E-20 | 4.53E-20 | 1.590 | 0.662 | 0.000 | -0.360 | -0.305 | -0.388 | -0.558 |
| C3 | 1.00E-20 | 4.53E-20 | 1.719 | 0.633 | 0.000 | -0.315 | -0.334 | -0.283 | -0.557 |
| JADE1 | 1.00E-20 | 4.53E-20 | 1.154 | 0.764 | 0.000 | -0.918 | -0.454 | -0.540 | -0.557 |
| SOD1 | 1.00E-20 | 4.53E-20 | 2.730 | 0.435 | 0.000 | -0.313 | -0.352 | -0.369 | -0.556 |
| FAXDC2 | 1.00E-20 | 4.53E-20 | 2.978 | 0.395 | 0.000 | -0.385 | -0.355 | -0.365 | -0.555 |
| HNRNPL | 1.00E-20 | 4.53E-20 | 2.419 | 0.490 | 0.000 | -0.335 | -0.315 | -0.336 | -0.554 |
| PRIMPOL | 1.00E-20 | 4.53E-20 | 1.046 | 0.790 | 0.000 | -0.337 | -0.382 | -0.439 | -0.554 |
| ENTPD6 | 1.00E-20 | 4.53E-20 | 1.672 | 0.643 | 0.000 | -0.336 | -0.311 | -0.324 | -0.554 |
| CAPN3 | 1.00E-20 | 4.53E-20 | 1.310 | 0.727 | 0.000 | -0.381 | -0.382 | -0.506 | -0.553 |
| C16orf58 | 1.00E-20 | 4.53E-20 | 1.966 | 0.579 | 0.000 | -0.305 | -0.287 | -0.287 | -0.552 |
| ABCA9 | 1.00E-20 | 4.53E-20 | 0.928 | 0.819 | 0.000 | -0.604 | -0.385 | -0.649 | -0.552 |
| FLNA | 1.00E-20 | 4.53E-20 | 1.203 | 0.752 | 0.000 | -0.329 | -0.337 | -0.344 | -0.551 |
| NPHP3 | 1.00E-20 | 4.53E-20 | 1.254 | 0.740 | 0.000 | -0.566 | -0.457 | -0.433 | -0.551 |
| ARFGAP3 | 1.00E-20 | 4.53E-20 | 1.687 | 0.640 | 0.000 | -0.349 | -0.371 | -0.364 | -0.549 |
| GPC3 | 1.00E-20 | 4.53E-20 | 0.551 | 0.908 | 0.000 | -0.839 | -0.408 | -0.631 | -0.546 |
| COL6A2 | 1.00E-20 | 4.53E-20 | 2.458 | 0.483 | 0.000 | -0.390 | -0.364 | -0.383 | -0.546 |
| CTSF | 1.00E-20 | 4.53E-20 | 2.024 | 0.568 | 0.000 | -0.448 | -0.371 | -0.447 | -0.545 |
| KRT35 | 0.003617 | 0.008231 | 1.805 | 0.614 | 0.000 | -0.384 | -0.405 | -0.601 | -0.544 |
| KLHL31 | 1.00E-20 | 4.53E-20 | 1.278 | 0.734 | 0.000 | -0.461 | -0.304 | -0.461 | -0.543 |
| RYR3 | 1.00E-20 | 4.53E-20 | 1.498 | 0.683 | 0.000 | -0.494 | -0.318 | -0.601 | -0.543 |
| PIP5K1C | 1.00E-20 | 4.53E-20 | 1.884 | 0.597 | 0.000 | -0.309 | -0.294 | -0.372 | -0.543 |
| RSF1 | 1.00E-20 | 4.53E-20 | 1.667 | 0.644 | 0.000 | -0.370 | -0.412 | -0.399 | -0.542 |
| POU3F3 | 1.00E-20 | 4.53E-20 | 2.690 | 0.442 | 0.000 | -0.672 | -0.386 | -0.509 | -0.542 |
| PLSCR4 | 1.00E-20 | 4.53E-20 | 1.975 | 0.578 | 0.000 | -0.325 | -0.343 | -0.351 | -0.542 |
| CAP2 | 1.00E-20 | 4.53E-20 | 2.245 | 0.523 | 0.000 | -0.306 | -0.317 | -0.327 | -0.542 |
| MOGAT1 | 1.00E-20 | 4.53E-20 | 0.543 | 0.909 | 0.000 | -0.746 | -0.441 | -0.742 | -0.541 |
| NTAN1 | 1.00E-20 | 4.53E-20 | 1.534 | 0.674 | 0.000 | -0.317 | -0.326 | -0.325 | -0.541 |
| ARHGEF10 | 1.00E-20 | 4.53E-20 | 2.528 | 0.470 | 0.000 | -1.066 | -0.457 | -0.625 | -0.540 |
| USP33 | 1.00E-20 | 4.53E-20 | 2.706 | 0.439 | 0.000 | -0.419 | -0.346 | -0.432 | -0.540 |
| ZNF862 | 1.00E-20 | 4.53E-20 | 1.953 | 0.582 | 0.000 | -0.425 | -0.328 | -0.362 | -0.539 |
| SLC25A16 | 1.00E-20 | 4.53E-20 | 0.158 | 0.984 | 0.000 | -0.330 | -0.384 | -0.407 | -0.539 |
| THBS3 | 1.00E-20 | 4.53E-20 | 1.084 | 0.781 | 0.000 | -0.357 | -0.337 | -0.359 | -0.539 |
| TLE4 | 1.00E-20 | 4.53E-20 | 0.662 | 0.882 | 0.000 | -0.390 | -0.353 | -0.487 | -0.539 |
| TSPOAP1 | 1.00E-20 | 4.53E-20 | 0.503 | 0.918 | 0.000 | -0.542 | -0.333 | -0.398 | -0.537 |
| CRTAP | 1.00E-20 | 4.53E-20 | 2.567 | 0.463 | 0.000 | -0.326 | -0.353 | -0.358 | -0.537 |
| CYB561A3 | 1.00E-20 | 4.53E-20 | 0.639 | 0.888 | 0.000 | -0.299 | -0.298 | -0.312 | -0.535 |
| CALB2 | 1.00E-20 | 4.53E-20 | 1.167 | 0.761 | 0.000 | -0.606 | -0.306 | -0.362 | -0.534 |
| TIMP4 | 1.00E-20 | 4.53E-20 | 1.218 | 0.749 | 0.000 | -1.032 | -0.493 | -0.795 | -0.534 |
| ACVR2A | 1.00E-20 | 4.53E-20 | 2.433 | 0.488 | 0.000 | -0.310 | -0.396 | -0.415 | -0.534 |
| TM4SF1 | 1.00E-20 | 4.53E-20 | 2.872 | 0.412 | 0.000 | -0.328 | -0.303 | -0.347 | -0.533 |
| MTA1 | 1.00E-20 | 4.53E-20 | 2.292 | 0.514 | 0.000 | -0.315 | -0.334 | -0.353 | -0.532 |
| SULT1A2 | 1.00E-20 | 4.53E-20 | 0.052 | 0.997 | 0.000 | -0.337 | -0.340 | -0.363 | -0.532 |
| TXNDC15 | 1.00E-20 | 4.53E-20 | 1.371 | 0.712 | 0.000 | -0.329 | -0.339 | -0.345 | -0.530 |
| KRT8 | 1.00E-20 | 4.53E-20 | 2.653 | 0.448 | 0.000 | -0.325 | -0.342 | -0.370 | -0.528 |
| RHOBTB1 | 1.00E-20 | 4.53E-20 | 1.625 | 0.654 | 0.000 | -0.418 | -0.362 | -0.446 | -0.527 |
| ZNF211 | 1.00E-20 | 4.53E-20 | 2.198 | 0.532 | 0.000 | -0.505 | -0.342 | -0.420 | -0.527 |
| GPATCH8 | 1.00E-20 | 4.53E-20 | 2.005 | 0.571 | 0.000 | -0.419 | -0.354 | -0.428 | -0.526 |
| PLCH2 | 1.00E-20 | 4.53E-20 | 1.687 | 0.640 | 0.000 | -0.408 | -0.338 | -0.414 | -0.525 |
| HMGCL | 1.00E-20 | 4.53E-20 | 2.892 | 0.409 | 0.000 | -0.379 | -0.314 | -0.307 | -0.525 |
| PHF2 | 1.00E-20 | 4.53E-20 | 0.685 | 0.877 | 0.000 | -0.338 | -0.369 | -0.407 | -0.525 |
| ACADM | 5.75E-07 | 2.44E-06 | 0.644 | 0.886 | 0.000 | -0.329 | -0.318 | -0.319 | -0.524 |
| PHF3 | 1.00E-20 | 4.53E-20 | 0.991 | 0.803 | 0.000 | -0.321 | -0.367 | -0.407 | -0.523 |
| IDH3A | 1.00E-20 | 4.53E-20 | 0.703 | 0.872 | 0.000 | -0.448 | -0.301 | -0.407 | -0.520 |
| OCA2 | 1.00E-20 | 4.53E-20 | 1.654 | 0.647 | 0.000 | -0.536 | -0.350 | -0.478 | -0.520 |
| SNCG | 1.00E-20 | 4.53E-20 | 2.813 | 0.421 | 0.000 | -0.739 | -0.328 | -0.314 | -0.519 |
| NRTN | 1.00E-20 | 4.53E-20 | 1.458 | 0.692 | 0.000 | -0.500 | -0.392 | -0.391 | -0.519 |
| CALCRL | 1.15E-06 | 4.51E-06 | 1.360 | 0.715 | 0.000 | -0.329 | -0.289 | -0.301 | -0.518 |
| IKZF5 | 1.00E-20 | 4.53E-20 | 2.635 | 0.451 | 0.000 | -0.328 | -0.344 | -0.381 | -0.516 |
| MOSPD2 | 1.00E-20 | 4.53E-20 | 2.087 | 0.554 | 0.000 | -0.413 | -0.371 | -0.459 | -0.515 |
| LEPROT | 1.00E-20 | 4.53E-20 | 1.214 | 0.750 | 0.000 | -0.296 | -0.352 | -0.354 | -0.515 |
| SBF1 | 1.00E-20 | 4.53E-20 | 1.390 | 0.708 | 0.000 | -0.296 | -0.336 | -0.436 | -0.515 |
| NFE2L1 | 1.00E-20 | 4.53E-20 | 1.050 | 0.789 | 0.000 | -0.322 | -0.344 | -0.394 | -0.515 |
| DSTYK | 1.00E-20 | 4.53E-20 | 1.220 | 0.748 | 0.000 | -0.319 | -0.371 | -0.427 | -0.514 |
| UCHL1 | 1.00E-20 | 4.53E-20 | 2.828 | 0.419 | 0.000 | -0.785 | -0.336 | -0.378 | -0.514 |
| CCDC146 | 1.00E-20 | 4.53E-20 | 1.233 | 0.745 | 0.000 | -0.589 | -0.455 | -0.610 | -0.513 |
| MYOF | 1.00E-20 | 4.53E-20 | 2.774 | 0.428 | 0.000 | -0.432 | -0.370 | -0.436 | -0.512 |
| GSAP | 1.00E-20 | 4.53E-20 | 0.428 | 0.934 | 0.000 | -0.345 | -0.350 | -0.386 | -0.511 |
| PLEKHA1 | 1.00E-20 | 4.53E-20 | 1.431 | 0.698 | 0.000 | -0.314 | -0.389 | -0.389 | -0.511 |
| GIGYF1 | 1.00E-20 | 4.53E-20 | 2.119 | 0.548 | 0.000 | -0.398 | -0.353 | -0.502 | -0.509 |
| PCK1 | 1.00E-20 | 4.53E-20 | 0.159 | 0.984 | 0.000 | -0.822 | -0.424 | -0.706 | -0.509 |
| ZFPM2 | 1.00E-20 | 4.53E-20 | 0.432 | 0.934 | 0.000 | -0.591 | -0.441 | -0.580 | -0.509 |
| ESPN | 1.00E-20 | 4.53E-20 | 1.796 | 0.616 | 0.000 | -0.368 | -0.311 | -0.331 | -0.508 |
| MMS19 | 1.00E-20 | 4.53E-20 | 0.998 | 0.802 | 0.000 | -0.299 | -0.293 | -0.364 | -0.506 |
| TENT2 | 1.00E-20 | 4.53E-20 | 0.909 | 0.823 | 0.000 | -0.399 | -0.425 | -0.531 | -0.505 |
| LRRC37A | 1.00E-20 | 4.53E-20 | 2.443 | 0.486 | 0.000 | -0.377 | -0.309 | -0.347 | -0.505 |
| POLR2M | 1.00E-20 | 4.53E-20 | 2.757 | 0.431 | 0.000 | -0.314 | -0.340 | -0.368 | -0.504 |
| LIFR | 1.00E-20 | 4.53E-20 | 1.906 | 0.592 | 0.000 | -0.880 | -0.510 | -0.669 | -0.504 |
| OAZ2 | 1.00E-20 | 4.53E-20 | 1.887 | 0.596 | 0.000 | -0.332 | -0.300 | -0.358 | -0.503 |
| ME3 | 1.15E-06 | 4.51E-06 | 2.246 | 0.523 | 0.000 | -0.336 | -0.288 | -0.329 | -0.503 |
| KIDINS220 | 1.00E-20 | 4.53E-20 | 2.738 | 0.434 | 0.000 | -0.709 | -0.320 | -0.415 | -0.502 |
| PHF10 | 1.00E-20 | 4.53E-20 | 2.646 | 0.449 | 0.000 | -0.353 | -0.373 | -0.412 | -0.502 |
| RALGAPA1 | 1.00E-20 | 4.53E-20 | 1.122 | 0.772 | 0.000 | -0.485 | -0.394 | -0.475 | -0.502 |
| DEPTOR | 1.00E-20 | 4.53E-20 | 0.269 | 0.966 | 0.000 | -0.356 | -0.396 | -0.381 | -0.501 |
| GFRA3 | 1.00E-20 | 4.53E-20 | 1.934 | 0.586 | 0.000 | -0.576 | -0.387 | -0.471 | -0.501 |
| C16orf45 | 1.00E-20 | 4.53E-20 | 2.601 | 0.457 | 0.000 | -0.359 | -0.356 | -0.366 | -0.500 |
| AMOTL2 | 1.00E-20 | 4.53E-20 | 1.925 | 0.588 | 0.000 | -0.298 | -0.338 | -0.366 | -0.500 |
| ZNF462 | 1.00E-20 | 4.53E-20 | 0.562 | 0.905 | 0.000 | -0.351 | -0.346 | -0.382 | -0.500 |
| NBEA | 1.00E-20 | 4.53E-20 | 2.624 | 0.453 | 0.000 | -0.927 | -0.361 | -0.500 | -0.499 |
| AIFM2 | 1.00E-20 | 4.53E-20 | 1.769 | 0.622 | 0.000 | -0.584 | -0.322 | -0.432 | -0.499 |
| ZNF43 | 1.00E-20 | 4.53E-20 | 1.338 | 0.720 | 0.000 | -0.474 | -0.365 | -0.419 | -0.499 |
| BAMBI | 1.00E-20 | 4.53E-20 | 1.498 | 0.683 | 0.000 | -0.490 | -0.348 | -0.496 | -0.498 |
| SPPL2B | 1.00E-20 | 4.53E-20 | 0.009 | 1.000 | 0.000 | -0.371 | -0.289 | -0.327 | -0.498 |
| FBLN2 | 1.00E-20 | 4.53E-20 | 2.348 | 0.503 | 0.000 | -0.351 | -0.359 | -0.366 | -0.497 |
| C6orf48 | 1.00E-20 | 4.53E-20 | 2.051 | 0.562 | 0.000 | -0.318 | -0.338 | -0.356 | -0.497 |
| BCL7A | 1.00E-20 | 4.53E-20 | 2.384 | 0.497 | 0.000 | -0.564 | -0.343 | -0.512 | -0.497 |
| SPOCK1 | 1.00E-20 | 4.53E-20 | 0.384 | 0.943 | 0.000 | -0.422 | -0.363 | -0.500 | -0.497 |
| COCH | 1.00E-20 | 4.53E-20 | 1.298 | 0.730 | 0.000 | -1.014 | -0.562 | -0.740 | -0.496 |
| RABEP1 | 1.00E-20 | 4.53E-20 | 2.005 | 0.571 | 0.000 | -0.328 | -0.331 | -0.354 | -0.496 |
| RABL2A | 1.00E-20 | 4.53E-20 | 1.045 | 0.790 | 0.000 | -0.335 | -0.315 | -0.394 | -0.496 |
| FUK | 1.00E-20 | 4.53E-20 | 1.158 | 0.763 | 0.000 | -0.315 | -0.286 | -0.299 | -0.496 |
| H2AFJ | 1.00E-20 | 4.53E-20 | 2.109 | 0.550 | 0.000 | -0.397 | -0.381 | -0.415 | -0.494 |
| TRIM52 | 1.00E-20 | 4.53E-20 | 1.803 | 0.614 | 0.000 | -0.389 | -0.361 | -0.482 | -0.494 |
| S100A13 | 1.00E-20 | 4.53E-20 | 2.400 | 0.494 | 0.000 | -0.339 | -0.310 | -0.309 | -0.493 |
| YIPF5 | 1.00E-20 | 4.53E-20 | 1.455 | 0.693 | 0.000 | -0.347 | -0.381 | -0.424 | -0.493 |
| ATG14 | 1.00E-20 | 4.53E-20 | 2.416 | 0.491 | 0.000 | -0.335 | -0.395 | -0.395 | -0.493 |
| 2-Sep | 1.00E-20 | 4.53E-20 | 1.829 | 0.609 | 0.000 | -0.597 | -0.353 | -0.356 | -0.493 |
| KRT33A | 1.15E-06 | 4.51E-06 | 1.070 | 0.784 | 0.000 | -0.499 | -0.348 | -0.539 | -0.491 |
| SENP6 | 1.00E-20 | 4.53E-20 | 2.039 | 0.564 | 0.000 | -0.554 | -0.397 | -0.489 | -0.491 |
| PLCB4 | 1.00E-20 | 4.53E-20 | 2.608 | 0.456 | 0.000 | -0.888 | -0.533 | -0.687 | -0.490 |
| SLC17A5 | 1.00E-20 | 4.53E-20 | 2.461 | 0.482 | 0.000 | -0.330 | -0.341 | -0.310 | -0.489 |
| FAM162B | 1.00E-20 | 4.53E-20 | 2.844 | 0.416 | 0.000 | -0.449 | -0.339 | -0.412 | -0.489 |
| LYRM7 | 1.00E-20 | 4.53E-20 | 2.759 | 0.430 | 0.000 | -0.357 | -0.388 | -0.375 | -0.488 |
| ZNF419 | 1.00E-20 | 4.53E-20 | 1.847 | 0.605 | 0.000 | -0.501 | -0.384 | -0.528 | -0.487 |
| SMAD3 | 1.00E-20 | 4.53E-20 | 1.481 | 0.687 | 0.000 | -0.312 | -0.346 | -0.438 | -0.487 |
| MSL1 | 1.00E-20 | 4.53E-20 | 0.260 | 0.967 | 0.000 | -0.310 | -0.336 | -0.351 | -0.486 |
| MEGF9 | 1.00E-20 | 4.53E-20 | 2.368 | 0.500 | 0.000 | -0.430 | -0.446 | -0.578 | -0.486 |
| ASMTL | 1.00E-20 | 4.53E-20 | 1.305 | 0.728 | 0.000 | -0.447 | -0.364 | -0.339 | -0.486 |
| ZNF141 | 1.00E-20 | 4.53E-20 | 2.557 | 0.465 | 0.000 | -0.340 | -0.292 | -0.432 | -0.484 |
| GTF2H2 | 1.00E-20 | 4.53E-20 | 0.361 | 0.948 | 0.000 | -0.359 | -0.359 | -0.361 | -0.483 |
| NEURL1B | 1.00E-20 | 4.53E-20 | 2.507 | 0.474 | 0.000 | -0.354 | -0.351 | -0.431 | -0.482 |
| PRXL2A | 1.00E-20 | 4.53E-20 | 1.443 | 0.696 | 0.000 | -0.354 | -0.370 | -0.388 | -0.482 |
| MTF2 | 1.00E-20 | 4.53E-20 | 1.744 | 0.627 | 0.000 | -0.330 | -0.304 | -0.341 | -0.480 |
| ADCY6 | 1.00E-20 | 4.53E-20 | 1.440 | 0.696 | 0.000 | -0.576 | -0.321 | -0.478 | -0.480 |
| UPB1 | 1.00E-20 | 4.53E-20 | 2.045 | 0.563 | 0.000 | -0.828 | -0.294 | -0.310 | -0.478 |
| MAFK | 1.00E-20 | 4.53E-20 | 2.756 | 0.431 | 0.000 | -0.305 | -0.336 | -0.377 | -0.478 |
| CAMTA1 | 1.00E-20 | 4.53E-20 | 2.759 | 0.430 | 0.000 | -0.432 | -0.375 | -0.416 | -0.477 |
| TOMM7 | 1.00E-20 | 4.53E-20 | 2.681 | 0.444 | 0.000 | -0.299 | -0.345 | -0.346 | -0.477 |
| TRIM68 | 1.00E-20 | 4.53E-20 | 1.008 | 0.799 | 0.000 | -0.299 | -0.311 | -0.344 | -0.476 |
| TSKU | 1.00E-20 | 4.53E-20 | 0.793 | 0.851 | 0.000 | -0.320 | -0.332 | -0.341 | -0.476 |
| KIF3A | 1.00E-20 | 4.53E-20 | 1.963 | 0.580 | 0.000 | -0.312 | -0.299 | -0.352 | -0.475 |
| AFAP1 | 1.00E-20 | 4.53E-20 | 2.372 | 0.499 | 0.000 | -0.361 | -0.284 | -0.310 | -0.475 |
| SMIM10 | 1.00E-20 | 4.53E-20 | 1.470 | 0.689 | 0.000 | -0.459 | -0.302 | -0.392 | -0.475 |
| SGF29 | 1.00E-20 | 4.53E-20 | 2.675 | 0.444 | 0.000 | -0.308 | -0.313 | -0.299 | -0.475 |
| MTUS1 | 1.00E-20 | 4.53E-20 | 0.655 | 0.884 | 0.000 | -0.417 | -0.336 | -0.399 | -0.475 |
| COA5 | 1.00E-20 | 4.53E-20 | 1.822 | 0.610 | 0.000 | -0.315 | -0.310 | -0.310 | -0.475 |
| SCARB1 | 1.00E-20 | 4.53E-20 | 1.229 | 0.746 | 0.000 | -0.458 | -0.347 | -0.371 | -0.474 |
| BBS10 | 1.00E-20 | 4.53E-20 | 1.752 | 0.625 | 0.000 | -0.344 | -0.360 | -0.399 | -0.474 |
| TLCD1 | 1.00E-20 | 4.53E-20 | 2.567 | 0.463 | 0.000 | -0.377 | -0.375 | -0.399 | -0.474 |
| TPSAB1 | 0.00066 | 0.001745 | 1.817 | 0.611 | 0.000 | -0.355 | -0.324 | -0.273 | -0.473 |
| CTSH | 1.00E-20 | 4.53E-20 | 2.360 | 0.501 | 0.000 | -0.292 | -0.322 | -0.267 | -0.473 |
| SPART | 1.00E-20 | 4.53E-20 | 0.661 | 0.882 | 0.000 | -0.565 | -0.419 | -0.524 | -0.472 |
| GYG2 | 1.00E-20 | 4.53E-20 | 0.240 | 0.971 | 0.000 | -0.793 | -0.369 | -0.661 | -0.471 |
| MEDAG | 0.000103 | 0.000315 | 1.515 | 0.679 | 0.000 | -0.365 | -0.302 | -0.322 | -0.471 |
| USP11 | 1.00E-20 | 4.53E-20 | 1.929 | 0.587 | 0.000 | -0.341 | -0.315 | -0.345 | -0.471 |
| CCDC80 | 2.88E-06 | 1.09E-05 | 1.682 | 0.641 | 0.000 | -0.360 | -0.323 | -0.359 | -0.470 |
| RNF13 | 1.00E-20 | 4.53E-20 | 0.879 | 0.831 | 0.000 | -0.295 | -0.324 | -0.318 | -0.469 |
| ENY2 | 1.00E-20 | 4.53E-20 | 2.571 | 0.463 | 0.000 | -0.450 | -0.441 | -0.393 | -0.469 |
| OSBPL8 | 1.00E-20 | 4.53E-20 | 0.556 | 0.906 | 0.000 | -0.430 | -0.421 | -0.607 | -0.469 |
| PRB4 | 1.00E-20 | 4.53E-20 | 2.849 | 0.415 | 0.000 | -0.734 | -0.301 | -0.422 | -0.469 |
| CDC42EP3 | 1.00E-20 | 4.53E-20 | 0.542 | 0.910 | 0.000 | -0.294 | -0.317 | -0.322 | -0.469 |
| MED31 | 1.00E-20 | 4.53E-20 | 2.849 | 0.416 | 0.000 | -0.451 | -0.386 | -0.438 | -0.468 |
| VIPR2 | 1.00E-20 | 4.53E-20 | 2.058 | 0.560 | 0.000 | -0.280 | -0.295 | -0.287 | -0.468 |
| SLC44A1 | 1.00E-20 | 4.53E-20 | 2.159 | 0.540 | 0.000 | -0.359 | -0.344 | -0.334 | -0.467 |
| AMIGO2 | 5.18E-06 | 1.92E-05 | 0.298 | 0.960 | 0.000 | -0.338 | -0.299 | -0.308 | -0.467 |
| FNBP1 | 1.00E-20 | 4.53E-20 | 1.074 | 0.783 | 0.000 | -0.444 | -0.382 | -0.358 | -0.467 |
| TMEM263 | 1.00E-20 | 4.53E-20 | 0.777 | 0.855 | 0.000 | -0.313 | -0.314 | -0.321 | -0.467 |
| ECHDC3 | 1.00E-20 | 4.53E-20 | 1.062 | 0.786 | 0.000 | -0.330 | -0.325 | -0.330 | -0.467 |
| CCDC130 | 0.026129 | 0.048749 | 1.528 | 0.676 | 0.000 | -0.271 | -0.270 | -0.276 | -0.466 |
| APBA1 | 1.00E-20 | 4.53E-20 | 1.858 | 0.602 | 0.000 | -0.726 | -0.363 | -0.635 | -0.466 |
| ZNF738 | 1.00E-20 | 4.53E-20 | 2.108 | 0.550 | 0.000 | -0.397 | -0.351 | -0.396 | -0.465 |
| C11orf80 | 1.00E-20 | 4.53E-20 | 0.741 | 0.864 | 0.000 | -0.425 | -0.356 | -0.431 | -0.465 |
| FOSL2 | 1.00E-20 | 4.53E-20 | 1.885 | 0.597 | 0.000 | -0.296 | -0.347 | -0.409 | -0.464 |
| DLGAP4 | 1.00E-20 | 4.53E-20 | 2.676 | 0.444 | 0.000 | -0.340 | -0.318 | -0.347 | -0.463 |
| SHISA2 | 1.00E-20 | 4.53E-20 | 1.096 | 0.778 | 0.000 | -0.515 | -0.392 | -0.546 | -0.463 |
| NAP1L3 | 1.00E-20 | 4.53E-20 | 0.654 | 0.884 | 0.000 | -0.579 | -0.461 | -0.552 | -0.463 |
| TYSND1 | 1.00E-20 | 4.53E-20 | 0.956 | 0.812 | 0.000 | -0.444 | -0.363 | -0.517 | -0.462 |
| APCDD1 | 1.00E-20 | 4.53E-20 | 2.369 | 0.499 | 0.000 | -0.632 | -0.347 | -0.472 | -0.460 |
| RERG | 1.00E-20 | 4.53E-20 | 2.753 | 0.431 | 0.000 | -0.476 | -0.418 | -0.444 | -0.456 |
| RNF24 | 1.00E-20 | 4.53E-20 | 2.032 | 0.566 | 0.000 | -0.541 | -0.366 | -0.447 | -0.455 |
| F10 | 1.00E-20 | 4.53E-20 | 2.783 | 0.426 | 0.000 | -0.539 | -0.392 | -0.387 | -0.455 |
| SLCO4C1 | 1.00E-20 | 4.53E-20 | 1.602 | 0.659 | 0.000 | -1.051 | -0.369 | -0.481 | -0.452 |
| PNLDC1 | 1.00E-20 | 4.53E-20 | 1.457 | 0.692 | 0.000 | -0.978 | -0.363 | -0.607 | -0.451 |
| UGT2B11 | 1.00E-20 | 4.53E-20 | 0.959 | 0.811 | 0.000 | -0.495 | -0.314 | -0.418 | -0.450 |
| SHC2 | 1.00E-20 | 4.53E-20 | 1.055 | 0.788 | 0.000 | -0.597 | -0.341 | -0.366 | -0.449 |
| MICU3 | 1.00E-20 | 4.53E-20 | 1.958 | 0.581 | 0.000 | -0.656 | -0.365 | -0.543 | -0.448 |
| DZIP1 | 1.00E-20 | 4.53E-20 | 1.909 | 0.592 | 0.000 | -0.481 | -0.322 | -0.364 | -0.447 |
| FIGN | 1.00E-20 | 4.53E-20 | 1.015 | 0.798 | 0.000 | -0.778 | -0.413 | -0.467 | -0.446 |
| PDE6A | 1.00E-20 | 4.53E-20 | 1.746 | 0.627 | 0.000 | -0.774 | -0.391 | -0.425 | -0.444 |
| CDR2 | 1.00E-20 | 4.53E-20 | 0.827 | 0.843 | 0.000 | -0.397 | -0.319 | -0.468 | -0.443 |
| ANGPTL7 | 1.00E-20 | 4.53E-20 | 0.199 | 0.978 | 0.000 | -0.678 | -0.359 | -0.408 | -0.442 |
| FBP1 | 1.00E-20 | 4.53E-20 | 1.866 | 0.601 | 0.000 | -1.015 | -0.394 | -0.522 | -0.442 |
| TRARG1 | 1.15E-06 | 4.51E-06 | 1.292 | 0.731 | 0.000 | -0.524 | -0.293 | -0.450 | -0.441 |
| PDE3B | 0.00418 | 0.009402 | 2.001 | 0.572 | 0.000 | -0.416 | -0.264 | -0.468 | -0.441 |
| BCL2 | 1.00E-20 | 4.53E-20 | 1.907 | 0.592 | 0.000 | -0.467 | -0.305 | -0.418 | -0.440 |
| CNTNAP3 | 1.00E-20 | 4.53E-20 | 2.008 | 0.571 | 0.000 | -0.944 | -0.310 | -0.716 | -0.436 |
| C14orf180 | 1.00E-20 | 4.53E-20 | 0.134 | 0.988 | 0.000 | -0.536 | -0.299 | -0.316 | -0.434 |
| EGFL7 | 1.00E-20 | 4.53E-20 | 0.627 | 0.890 | 0.000 | -0.485 | -0.313 | -0.444 | -0.434 |
| ALDH6A1 | 1.00E-20 | 4.53E-20 | 1.208 | 0.751 | 0.000 | -0.805 | -0.400 | -0.596 | -0.433 |
| TUB | 1.00E-20 | 4.53E-20 | 1.953 | 0.582 | 0.000 | -0.465 | -0.353 | -0.485 | -0.433 |
| USP40 | 1.00E-20 | 4.53E-20 | 1.262 | 0.738 | 0.000 | -0.481 | -0.307 | -0.505 | -0.433 |
| CMYA5 | 1.00E-20 | 4.53E-20 | 2.387 | 0.496 | 0.000 | -0.850 | -0.311 | -0.461 | -0.432 |
| NUDT11 | 1.00E-20 | 4.53E-20 | 1.110 | 0.775 | 0.000 | -0.533 | -0.370 | -0.536 | -0.432 |
| ACVR1C | 1.00E-20 | 4.53E-20 | 0.573 | 0.903 | 0.000 | -0.641 | -0.300 | -0.457 | -0.430 |
| ROR1 | 1.00E-20 | 4.53E-20 | 2.823 | 0.420 | 0.000 | -0.560 | -0.403 | -0.372 | -0.430 |
| CEP290 | 1.00E-20 | 4.53E-20 | 2.188 | 0.534 | 0.000 | -0.470 | -0.317 | -0.474 | -0.429 |
| ZNF793 | 1.00E-20 | 4.53E-20 | 2.935 | 0.402 | 0.000 | -0.631 | -0.318 | -0.390 | -0.427 |
| LGALS12 | 1.00E-20 | 4.53E-20 | 0.568 | 0.904 | 0.000 | -0.540 | -0.306 | -0.506 | -0.426 |
| CEP126 | 1.00E-20 | 4.53E-20 | 1.707 | 0.635 | 0.000 | -0.479 | -0.319 | -0.415 | -0.425 |
| VIT | 1.00E-20 | 4.53E-20 | 2.349 | 0.503 | 0.000 | -0.788 | -0.463 | -0.574 | -0.423 |
| TOX3 | 1.00E-20 | 4.53E-20 | 1.627 | 0.653 | 0.000 | -0.782 | -0.393 | -0.696 | -0.423 |
| GNG7 | 1.00E-20 | 4.53E-20 | 2.239 | 0.524 | 0.000 | -0.501 | -0.346 | -0.392 | -0.422 |
| MMP16 | 1.00E-20 | 4.53E-20 | 1.328 | 0.723 | 0.000 | -0.567 | -0.324 | -0.416 | -0.420 |
| AFF1 | 1.00E-20 | 4.53E-20 | 2.714 | 0.438 | 0.000 | -0.571 | -0.314 | -0.444 | -0.420 |
| KIF21A | 1.00E-20 | 4.53E-20 | 1.777 | 0.620 | 0.000 | -0.452 | -0.433 | -0.647 | -0.420 |
| TMPRSS11E | 1.00E-20 | 4.53E-20 | 2.217 | 0.529 | 0.000 | -1.151 | -0.432 | -0.622 | -0.419 |
| DHRS2 | 5.75E-07 | 2.44E-06 | 0.415 | 0.937 | 0.000 | -0.614 | -0.303 | -0.366 | -0.419 |
| DMD | 1.00E-20 | 4.53E-20 | 1.999 | 0.573 | 0.000 | -0.840 | -0.331 | -0.723 | -0.419 |
| EDAR | 1.00E-20 | 4.53E-20 | 0.589 | 0.899 | 0.000 | -0.851 | -0.343 | -0.465 | -0.418 |
| ADGRF5 | 1.00E-20 | 4.53E-20 | 0.150 | 0.985 | 0.000 | -0.492 | -0.349 | -0.422 | -0.418 |
| EP400 | 5.75E-07 | 2.44E-06 | 1.798 | 0.615 | 0.000 | -0.319 | -0.335 | -0.482 | -0.417 |
| C2orf88 | 1.00E-20 | 4.53E-20 | 2.217 | 0.529 | 0.000 | -0.687 | -0.398 | -0.532 | -0.417 |
| GSTT2 | 6.33E-06 | 2.29E-05 | 1.045 | 0.790 | 0.000 | -0.462 | -0.430 | -0.660 | -0.416 |
| PPM1J | 1.00E-20 | 4.53E-20 | 2.100 | 0.552 | 0.000 | -0.496 | -0.329 | -0.434 | -0.415 |
| SLC27A2 | 1.00E-20 | 4.53E-20 | 0.158 | 0.984 | 0.000 | -1.036 | -0.420 | -0.647 | -0.415 |
| FZD8 | 1.00E-20 | 4.53E-20 | 0.698 | 0.874 | 0.000 | -0.544 | -0.416 | -0.583 | -0.414 |
| RPL31 | 1.00E-20 | 4.53E-20 | 1.898 | 0.594 | 0.000 | -0.884 | -0.332 | -0.483 | -0.414 |
| ZNF708 | 1.00E-20 | 4.53E-20 | 0.947 | 0.814 | 0.000 | -0.369 | -0.312 | -0.508 | -0.413 |
| SCAF11 | 1.00E-20 | 4.53E-20 | 2.326 | 0.508 | 0.000 | -0.418 | -0.325 | -0.502 | -0.413 |
| LTN1 | 1.00E-20 | 4.53E-20 | 1.106 | 0.776 | 0.000 | -0.332 | -0.399 | -0.516 | -0.411 |
| HRCT1 | 1.00E-20 | 4.53E-20 | 1.189 | 0.756 | 0.000 | -0.577 | -0.398 | -0.456 | -0.411 |
| OVGP1 | 1.00E-20 | 4.53E-20 | 1.440 | 0.696 | 0.000 | -0.589 | -0.316 | -0.390 | -0.410 |
| DKK1 | 1.00E-20 | 4.53E-20 | 1.382 | 0.710 | 0.000 | -0.944 | -0.362 | -0.737 | -0.407 |
| ACSM3 | 1.00E-20 | 4.53E-20 | 0.729 | 0.866 | 0.000 | -0.853 | -0.385 | -0.575 | -0.405 |
| PDZD2 | 1.00E-20 | 4.53E-20 | 0.819 | 0.845 | 0.000 | -0.508 | -0.335 | -0.522 | -0.405 |
| ZNF25 | 1.00E-20 | 4.53E-20 | 1.267 | 0.737 | 0.000 | -0.487 | -0.358 | -0.427 | -0.403 |
| SHE | 1.00E-20 | 4.53E-20 | 1.336 | 0.721 | 0.000 | -0.547 | -0.319 | -0.484 | -0.401 |
| CYS1 | 1.00E-20 | 4.53E-20 | 2.422 | 0.490 | 0.000 | -0.617 | -0.351 | -0.482 | -0.400 |
| MAP3K21 | 1.00E-20 | 4.53E-20 | 1.633 | 0.652 | 0.000 | -0.389 | -0.367 | -0.466 | -0.399 |
| RASD2 | 1.00E-20 | 4.53E-20 | 2.990 | 0.393 | 0.000 | -0.493 | -0.277 | -0.284 | -0.399 |
| METTL7B | 5.75E-07 | 2.44E-06 | 1.244 | 0.743 | 0.000 | -0.538 | -0.310 | -0.462 | -0.399 |
| CNTN3 | 1.00E-20 | 4.53E-20 | 2.224 | 0.527 | 0.000 | -0.560 | -0.387 | -0.470 | -0.399 |
| SEMA3B | 1.00E-20 | 4.53E-20 | 0.599 | 0.897 | 0.000 | -0.637 | -0.371 | -0.421 | -0.398 |
| FAM107A | 1.00E-20 | 4.53E-20 | 2.133 | 0.545 | 0.000 | -0.532 | -0.376 | -0.393 | -0.396 |
| CCL23 | 1.00E-20 | 4.53E-20 | 1.766 | 0.622 | 0.000 | -0.553 | -0.280 | -0.432 | -0.395 |
| C6 | 1.00E-20 | 4.53E-20 | 1.710 | 0.635 | 0.000 | -0.500 | -0.310 | -0.424 | -0.394 |
| PDZRN4 | 1.00E-20 | 4.53E-20 | 2.278 | 0.517 | 0.000 | -0.808 | -0.437 | -0.454 | -0.394 |
| LMO4 | 1.00E-20 | 4.53E-20 | 1.407 | 0.704 | 0.000 | -0.597 | -0.411 | -0.532 | -0.392 |
| TRAPPC12 | 1.00E-20 | 4.53E-20 | 1.961 | 0.581 | 0.000 | -0.466 | -0.304 | -0.361 | -0.392 |
| SELENOP | 1.00E-20 | 4.53E-20 | 1.597 | 0.660 | 0.000 | -0.539 | -0.408 | -0.475 | -0.392 |
| OGN | 1.00E-20 | 4.53E-20 | 1.324 | 0.723 | 0.000 | -0.594 | -0.467 | -0.483 | -0.391 |
| KMT2A | 1.00E-20 | 4.53E-20 | 1.477 | 0.688 | 0.000 | -0.578 | -0.320 | -0.417 | -0.390 |
| CRLS1 | 1.00E-20 | 4.53E-20 | 2.192 | 0.533 | 0.000 | -0.548 | -0.345 | -0.506 | -0.389 |
| ZNF514 | 1.00E-20 | 4.53E-20 | 1.067 | 0.785 | 0.000 | -0.601 | -0.408 | -0.449 | -0.388 |
| SLC45A4 | 1.00E-20 | 4.53E-20 | 1.543 | 0.672 | 0.000 | -0.677 | -0.349 | -0.527 | -0.388 |
| WASF2 | 1.00E-20 | 4.53E-20 | 2.777 | 0.427 | 0.000 | -0.499 | -0.354 | -0.466 | -0.387 |
| NDNF | 1.00E-20 | 4.53E-20 | 1.292 | 0.731 | 0.000 | -0.802 | -0.409 | -0.612 | -0.385 |
| RNF182 | 1.00E-20 | 4.53E-20 | 1.333 | 0.721 | 0.000 | -0.589 | -0.295 | -0.321 | -0.384 |
| MYOM2 | 1.00E-20 | 4.53E-20 | 2.623 | 0.453 | 0.000 | -0.652 | -0.362 | -0.451 | -0.384 |
| CENPV | 1.00E-20 | 4.53E-20 | 0.833 | 0.842 | 0.000 | -0.566 | -0.341 | -0.419 | -0.383 |
| FDX1 | 1.00E-20 | 4.53E-20 | 2.751 | 0.432 | 0.000 | -0.626 | -0.336 | -0.312 | -0.383 |
| TTC5 | 1.00E-20 | 4.53E-20 | 2.746 | 0.433 | 0.000 | -0.542 | -0.296 | -0.396 | -0.382 |
| UBIAD1 | 1.00E-20 | 4.53E-20 | 2.113 | 0.549 | 0.000 | -0.747 | -0.364 | -0.350 | -0.382 |
| ZNF850 | 1.00E-20 | 4.53E-20 | 2.476 | 0.480 | 0.000 | -0.703 | -0.315 | -0.417 | -0.381 |
| RPS23 | 1.00E-20 | 4.53E-20 | 0.972 | 0.808 | 0.000 | -0.469 | -0.381 | -0.399 | -0.381 |
| SLC26A3 | 1.00E-20 | 4.53E-20 | 1.480 | 0.687 | 0.000 | -0.870 | -0.368 | -0.457 | -0.380 |
| LRP5 | 1.00E-20 | 4.53E-20 | 2.539 | 0.468 | 0.000 | -0.363 | -0.316 | -0.505 | -0.379 |
| SGCD | 1.00E-20 | 4.53E-20 | 1.968 | 0.579 | 0.000 | -0.473 | -0.361 | -0.364 | -0.378 |
| FILIP1 | 1.00E-20 | 4.53E-20 | 0.940 | 0.816 | 0.000 | -0.593 | -0.324 | -0.407 | -0.378 |
| CCDC90B | 1.00E-20 | 4.53E-20 | 1.814 | 0.612 | 0.000 | -0.660 | -0.322 | -0.362 | -0.377 |
| RASEF | 1.00E-20 | 4.53E-20 | 1.589 | 0.662 | 0.000 | -0.541 | -0.334 | -0.454 | -0.376 |
| RGN | 1.00E-20 | 4.53E-20 | 1.970 | 0.579 | 0.000 | -0.529 | -0.308 | -0.413 | -0.376 |
| MSRB3 | 1.00E-20 | 4.53E-20 | 1.029 | 0.794 | 0.000 | -0.640 | -0.376 | -0.623 | -0.376 |
| SPATA6 | 1.00E-20 | 4.53E-20 | 2.015 | 0.569 | 0.000 | -0.549 | -0.394 | -0.612 | -0.376 |
| MGP | 1.00E-20 | 4.53E-20 | 0.891 | 0.828 | 0.000 | -0.713 | -0.377 | -0.569 | -0.375 |
| SERPINA5 | 1.00E-20 | 4.53E-20 | 1.792 | 0.617 | 0.000 | -0.599 | -0.427 | -0.583 | -0.374 |
| DIRAS3 | 1.00E-20 | 4.53E-20 | 0.674 | 0.879 | 0.000 | -0.598 | -0.333 | -0.357 | -0.373 |
| TOX2 | 1.00E-20 | 4.53E-20 | 1.677 | 0.642 | 0.000 | -0.493 | -0.333 | -0.399 | -0.372 |
| STBD1 | 1.00E-20 | 4.53E-20 | 1.703 | 0.636 | 0.000 | -0.596 | -0.299 | -0.511 | -0.371 |
| KRT32 | 0.00145 | 0.003591 | 0.221 | 0.974 | 0.000 | -0.465 | -0.299 | -0.469 | -0.371 |
| ZMYM5 | 1.00E-20 | 4.53E-20 | 2.320 | 0.509 | 0.000 | -0.545 | -0.352 | -0.388 | -0.371 |
| CCDC127 | 1.00E-20 | 4.53E-20 | 1.070 | 0.784 | 0.000 | -0.619 | -0.329 | -0.551 | -0.370 |
| DNAH8 | 1.00E-20 | 4.53E-20 | 0.701 | 0.873 | 0.000 | -0.783 | -0.347 | -0.391 | -0.369 |
| TCEAL7 | 1.00E-20 | 4.53E-20 | 2.193 | 0.533 | 0.000 | -0.568 | -0.379 | -0.418 | -0.369 |
| CGNL1 | 1.15E-06 | 4.51E-06 | 0.762 | 0.859 | 0.000 | -0.513 | -0.281 | -0.265 | -0.368 |
| ZMYND11 | 1.00E-20 | 4.53E-20 | 0.816 | 0.846 | 0.000 | -0.559 | -0.315 | -0.399 | -0.368 |
| TRMT13 | 1.00E-20 | 4.53E-20 | 2.267 | 0.519 | 0.000 | -0.544 | -0.411 | -0.515 | -0.367 |
| GALT | 1.00E-20 | 4.53E-20 | 2.100 | 0.552 | 0.000 | -0.565 | -0.292 | -0.414 | -0.367 |
| PRRG3 | 1.00E-20 | 4.53E-20 | 2.845 | 0.416 | 0.000 | -0.630 | -0.420 | -0.510 | -0.367 |
| CSN1S1 | 1.15E-06 | 4.51E-06 | 2.640 | 0.450 | 0.000 | -0.395 | -0.354 | -0.577 | -0.366 |
| TRIM55 | 1.00E-20 | 4.53E-20 | 0.902 | 0.825 | 0.000 | -0.597 | -0.280 | -0.274 | -0.364 |
| RASSF6 | 1.00E-20 | 4.53E-20 | 0.395 | 0.941 | 0.000 | -0.667 | -0.414 | -0.513 | -0.364 |
| PEX11A | 1.00E-20 | 4.53E-20 | 1.797 | 0.615 | 0.000 | -0.671 | -0.326 | -0.349 | -0.364 |
| TSPAN18 | 1.00E-20 | 4.53E-20 | 0.519 | 0.915 | 0.000 | -0.602 | -0.305 | -0.321 | -0.364 |
| KCNT2 | 1.00E-20 | 4.53E-20 | 0.705 | 0.872 | 0.000 | -0.569 | -0.327 | -0.481 | -0.364 |
| SPX | 1.00E-20 | 4.53E-20 | 1.980 | 0.577 | 0.000 | -0.551 | -0.439 | -0.490 | -0.364 |
| CFAP69 | 1.00E-20 | 4.53E-20 | 2.755 | 0.431 | 0.000 | -0.495 | -0.306 | -0.465 | -0.364 |
| ACOT4 | 1.00E-20 | 4.53E-20 | 2.396 | 0.494 | 0.000 | -0.665 | -0.304 | -0.346 | -0.363 |
| ZNF667 | 1.00E-20 | 4.53E-20 | 2.868 | 0.412 | 0.000 | -0.573 | -0.336 | -0.401 | -0.362 |
| HECTD4 | 1.15E-06 | 4.51E-06 | 2.746 | 0.432 | 0.000 | -0.522 | -0.274 | -0.298 | -0.361 |
| FAM200B | 1.00E-20 | 4.53E-20 | 2.110 | 0.550 | 0.000 | -0.647 | -0.335 | -0.433 | -0.361 |
| CCBE1 | 1.00E-20 | 4.53E-20 | 0.265 | 0.966 | 0.000 | -0.485 | -0.327 | -0.418 | -0.361 |
| GFRA1 | 1.00E-20 | 4.53E-20 | 2.867 | 0.413 | 0.000 | -0.658 | -0.317 | -0.498 | -0.360 |
| HEPACAM | 1.00E-20 | 4.53E-20 | 1.160 | 0.763 | 0.000 | -0.536 | -0.306 | -0.359 | -0.360 |
| SNX25 | 1.00E-20 | 4.53E-20 | 2.679 | 0.444 | 0.000 | -0.461 | -0.313 | -0.509 | -0.359 |
| PGR | 1.00E-20 | 4.53E-20 | 0.407 | 0.939 | 0.000 | -0.774 | -0.361 | -0.524 | -0.359 |
| ALX1 | 1.00E-20 | 4.53E-20 | 1.267 | 0.737 | 0.000 | -0.572 | -0.298 | -0.379 | -0.356 |
| LAMC2 | 1.00E-20 | 4.53E-20 | 2.432 | 0.488 | 0.000 | -0.479 | -0.288 | -0.433 | -0.355 |
| DNAJC7 | 1.00E-20 | 4.53E-20 | 0.519 | 0.915 | 0.000 | -0.435 | -0.351 | -0.613 | -0.354 |
| FN1 | 1.00E-20 | 4.53E-20 | 1.174 | 0.759 | 0.000 | -0.509 | -0.292 | -0.328 | -0.354 |
| HLCS | 1.00E-20 | 4.53E-20 | 0.394 | 0.941 | 0.000 | -0.645 | -0.325 | -0.360 | -0.354 |
| ARL9 | 1.00E-20 | 4.53E-20 | 0.546 | 0.909 | 0.000 | -0.398 | -0.317 | -0.495 | -0.354 |
| ACO1 | 0.003085 | 0.007122 | 0.240 | 0.971 | 0.000 | -0.469 | -0.312 | -0.387 | -0.354 |
| HEATR4 | 5.75E-07 | 2.44E-06 | 0.782 | 0.854 | 0.000 | -0.514 | -0.285 | -0.329 | -0.354 |
| MRVI1 | 1.00E-20 | 4.53E-20 | 1.254 | 0.740 | 0.000 | -0.511 | -0.427 | -0.578 | -0.353 |
| HBG1 | 5.75E-06 | 2.11E-05 | 0.157 | 0.984 | 0.000 | -0.451 | -0.375 | -0.623 | -0.353 |
| CYP1A1 | 1.00E-20 | 4.53E-20 | 0.054 | 0.997 | 0.000 | -0.755 | -0.401 | -0.635 | -0.352 |
| COLCA2 | 1.00E-20 | 4.53E-20 | 2.805 | 0.423 | 0.000 | -0.491 | -0.330 | -0.376 | -0.352 |
| KLHL13 | 1.00E-20 | 4.53E-20 | 1.995 | 0.573 | 0.000 | -0.524 | -0.336 | -0.474 | -0.352 |
| CHCHD2 | 1.00E-20 | 4.53E-20 | 1.017 | 0.797 | 0.000 | -0.496 | -0.408 | -0.413 | -0.352 |
| GON4L | 1.00E-20 | 4.53E-20 | 1.959 | 0.581 | 0.000 | -0.630 | -0.336 | -0.396 | -0.351 |
| OMD | 1.00E-20 | 4.53E-20 | 2.047 | 0.563 | 0.000 | -0.533 | -0.439 | -0.581 | -0.351 |
| HDGFL3 | 1.00E-20 | 4.53E-20 | 2.116 | 0.549 | 0.000 | -0.486 | -0.316 | -0.426 | -0.351 |
| ZNF263 | 1.00E-20 | 4.53E-20 | 2.751 | 0.432 | 0.000 | -0.493 | -0.328 | -0.331 | -0.351 |
| THSD7A | 1.00E-20 | 4.53E-20 | 1.365 | 0.714 | 0.000 | -0.594 | -0.330 | -0.444 | -0.350 |
| EMP1 | 1.00E-20 | 4.53E-20 | 0.348 | 0.951 | 0.000 | -0.489 | -0.294 | -0.422 | -0.350 |
| EFHC1 | 1.00E-20 | 4.53E-20 | 2.724 | 0.436 | 0.000 | -0.481 | -0.292 | -0.391 | -0.349 |
| FADS3 | 1.00E-20 | 4.53E-20 | 0.655 | 0.884 | 0.000 | -0.493 | -0.345 | -0.525 | -0.349 |
| FBXO22 | 1.00E-20 | 4.53E-20 | 2.596 | 0.458 | 0.000 | -0.518 | -0.377 | -0.380 | -0.348 |
| ELMSAN1 | 1.00E-20 | 4.53E-20 | 1.792 | 0.617 | 0.000 | -0.623 | -0.324 | -0.485 | -0.348 |
| OTUD3 | 1.00E-20 | 4.53E-20 | 1.592 | 0.661 | 0.000 | -0.414 | -0.344 | -0.490 | -0.347 |
| ZNF365 | 1.00E-20 | 4.53E-20 | 1.834 | 0.608 | 0.000 | -0.636 | -0.372 | -0.398 | -0.347 |
| TRIM44 | 1.00E-20 | 4.53E-20 | 2.689 | 0.442 | 0.000 | -0.531 | -0.309 | -0.361 | -0.346 |
| ZNF609 | 1.00E-20 | 4.53E-20 | 0.855 | 0.836 | 0.000 | -0.528 | -0.317 | -0.412 | -0.344 |
| RARRES1 | 0.000455 | 0.001237 | 0.532 | 0.912 | 0.000 | -0.521 | -0.372 | -0.380 | -0.343 |
| SEMA6D | 3.45E-06 | 1.30E-05 | 1.404 | 0.705 | 0.000 | -0.527 | -0.275 | -0.379 | -0.343 |
| ETFA | 1.00E-20 | 4.53E-20 | 2.213 | 0.529 | 0.000 | -0.524 | -0.354 | -0.622 | -0.342 |
| SMARCA1 | 1.00E-20 | 4.53E-20 | 2.922 | 0.404 | 0.000 | -0.603 | -0.378 | -0.492 | -0.342 |
| TDRD6 | 1.00E-20 | 4.53E-20 | 1.958 | 0.581 | 0.000 | -0.464 | -0.286 | -0.531 | -0.342 |
| C1orf52 | 1.00E-20 | 4.53E-20 | 2.391 | 0.495 | 0.000 | -0.475 | -0.315 | -0.460 | -0.341 |
| CELF6 | 1.00E-20 | 4.53E-20 | 1.001 | 0.801 | 0.000 | -0.479 | -0.280 | -0.348 | -0.340 |
| WDR5B | 1.00E-20 | 4.53E-20 | 2.130 | 0.546 | 0.000 | -0.410 | -0.323 | -0.484 | -0.340 |
| CLUAP1 | 1.00E-20 | 4.53E-20 | 2.251 | 0.522 | 0.000 | -0.545 | -0.314 | -0.389 | -0.339 |
| LAMA4 | 1.00E-20 | 4.53E-20 | 1.222 | 0.748 | 0.000 | -0.503 | -0.310 | -0.440 | -0.336 |
| PADI3 | 0.0014 | 0.003482 | 0.747 | 0.862 | 0.000 | -0.465 | -0.271 | -0.366 | -0.335 |
| ASPN | 1.00E-20 | 4.53E-20 | 2.946 | 0.400 | 0.000 | -0.678 | -0.477 | -0.611 | -0.335 |
| LGI1 | 1.00E-20 | 4.53E-20 | 1.428 | 0.699 | 0.000 | -0.661 | -0.295 | -0.277 | -0.334 |
| RPA1 | 1.00E-20 | 4.53E-20 | 0.400 | 0.940 | 0.000 | -0.507 | -0.321 | -0.333 | -0.333 |
| NUP88 | 1.00E-20 | 4.53E-20 | 2.677 | 0.444 | 0.000 | -0.630 | -0.326 | -0.372 | -0.332 |
| STRAP | 1.00E-20 | 4.53E-20 | 0.529 | 0.913 | 0.000 | -0.497 | -0.297 | -0.310 | -0.332 |
| CCDC186 | 1.00E-20 | 4.53E-20 | 2.484 | 0.478 | 0.000 | -0.316 | -0.333 | -0.517 | -0.332 |
| ZNF585A | 1.00E-20 | 4.53E-20 | 2.020 | 0.568 | 0.000 | -0.516 | -0.295 | -0.331 | -0.328 |
| PRG4 | 0.001293 | 0.003231 | 1.457 | 0.692 | 0.000 | -0.406 | -0.334 | -0.506 | -0.326 |
| PER2 | 1.00E-20 | 4.53E-20 | 0.377 | 0.945 | 0.000 | -0.570 | -0.322 | -0.354 | -0.326 |
| OLA1 | 1.00E-20 | 4.53E-20 | 2.347 | 0.503 | 0.000 | -0.699 | -0.366 | -0.445 | -0.324 |
| MATN4 | 1.00E-20 | 4.53E-20 | 2.313 | 0.510 | 0.000 | -0.588 | -0.348 | -0.322 | -0.324 |
| CAMKK2 | 1.00E-20 | 4.53E-20 | 2.560 | 0.465 | 0.000 | -0.475 | -0.323 | -0.341 | -0.321 |
| KALRN | 1.00E-20 | 4.53E-20 | 2.682 | 0.443 | 0.000 | -0.479 | -0.284 | -0.293 | -0.321 |
| EFHB | 1.00E-20 | 4.53E-20 | 0.994 | 0.803 | 0.000 | -0.506 | -0.300 | -0.374 | -0.319 |
| LZTFL1 | 1.00E-20 | 4.53E-20 | 2.515 | 0.473 | 0.000 | -0.476 | -0.285 | -0.334 | -0.318 |
| NEGR1 | 1.00E-20 | 4.53E-20 | 0.725 | 0.867 | 0.000 | -0.637 | -0.350 | -0.532 | -0.318 |
| KRTAP19-1 | 0.006543 | 0.014036 | 1.439 | 0.696 | 0.000 | -0.497 | -0.339 | -0.606 | -0.317 |
| RBBP4 | 1.00E-20 | 4.53E-20 | 2.795 | 0.424 | 0.000 | -0.475 | -0.318 | -0.315 | -0.316 |
| C12orf66 | 1.00E-20 | 4.53E-20 | 2.744 | 0.433 | 0.000 | -0.485 | -0.326 | -0.396 | -0.315 |
| NUDT13 | 1.00E-20 | 4.53E-20 | 0.305 | 0.959 | 0.000 | -0.466 | -0.307 | -0.444 | -0.314 |
| APOC2 | 1.00E-20 | 4.53E-20 | 1.212 | 0.750 | 0.000 | -0.463 | -0.303 | -0.335 | -0.314 |
| LEAP2 | 1.00E-20 | 4.53E-20 | 0.130 | 0.988 | 0.000 | -0.482 | -0.286 | -0.449 | -0.314 |
| NUMA1 | 0.011118 | 0.022632 | 1.053 | 0.788 | 0.000 | -0.473 | -0.312 | -0.361 | -0.313 |
| ATRX | 1.00E-20 | 4.53E-20 | 2.825 | 0.419 | 0.000 | -0.528 | -0.319 | -0.498 | -0.313 |
| CCDC32 | 1.00E-20 | 4.53E-20 | 0.849 | 0.838 | 0.000 | -0.417 | -0.301 | -0.467 | -0.312 |
| SESN1 | 1.00E-20 | 4.53E-20 | 2.589 | 0.459 | 0.000 | -0.509 | -0.305 | -0.354 | -0.311 |
| FBLN1 | 1.00E-20 | 4.53E-20 | 1.254 | 0.740 | 0.000 | -0.587 | -0.335 | -0.428 | -0.308 |
| VTI1A | 5.75E-07 | 2.44E-06 | 2.342 | 0.505 | 0.000 | -0.532 | -0.315 | -0.439 | -0.308 |
| CADPS | 1.00E-20 | 4.53E-20 | 1.248 | 0.741 | 0.000 | -0.651 | -0.335 | -0.444 | -0.304 |
| HNRNPU | 1.00E-20 | 4.53E-20 | 0.693 | 0.875 | 0.000 | -0.527 | -0.293 | -0.387 | -0.302 |
| SERTM1 | 1.00E-20 | 4.53E-20 | 0.880 | 0.830 | 0.000 | -0.708 | -0.388 | -0.280 | -0.302 |
| AGT | 1.00E-20 | 4.53E-20 | 2.464 | 0.482 | 0.000 | -0.739 | -0.365 | -0.324 | -0.302 |
| SYNJ2BP | 1.00E-20 | 4.53E-20 | 1.676 | 0.642 | 0.000 | -0.379 | -0.313 | -0.469 | -0.301 |
| CD55 | 5.75E-06 | 2.11E-05 | 0.738 | 0.864 | 0.000 | -0.527 | -0.281 | -0.282 | -0.301 |
| TMF1 | 1.00E-20 | 4.53E-20 | 2.715 | 0.438 | 0.000 | -0.492 | -0.284 | -0.367 | -0.300 |
| BEAN1 | 1.00E-20 | 4.53E-20 | 2.430 | 0.488 | 0.000 | -0.646 | -0.312 | -0.335 | -0.297 |
| VPS8 | 1.00E-20 | 4.53E-20 | 2.197 | 0.532 | 0.000 | -0.353 | -0.287 | -0.488 | -0.293 |
| PTGFR | 5.75E-07 | 2.44E-06 | 1.920 | 0.589 | 0.000 | -0.371 | -0.384 | -0.552 | -0.293 |
| EPN2 | 5.75E-06 | 2.11E-05 | 2.677 | 0.444 | 0.000 | -0.577 | -0.275 | -0.277 | -0.286 |
| SH3D19 | 1.15E-06 | 4.51E-06 | 2.042 | 0.564 | 0.000 | -0.493 | -0.296 | -0.473 | -0.285 |
| OSGIN2 | 1.00E-20 | 4.53E-20 | 2.161 | 0.540 | 0.000 | -0.469 | -0.320 | -0.414 | -0.282 |
| CRCP | 2.30E-06 | 8.92E-06 | 2.554 | 0.466 | 0.000 | -0.467 | -0.297 | -0.301 | -0.280 |
| UBE2Q1 | 0.000115 | 0.000349 | 2.400 | 0.494 | 0.000 | -0.501 | -0.272 | -0.298 | -0.277 |
| ARFGEF1 | 0.000169 | 0.000494 | 2.653 | 0.448 | 0.000 | -0.493 | -0.267 | -0.269 | -0.276 |
| FAM214A | 1.15E-06 | 4.51E-06 | 2.198 | 0.532 | 0.000 | 0.464 | 0.303 | 0.402 | 0.266 |
| CCDC102B | 1.15E-06 | 4.51E-06 | 1.751 | 0.626 | 0.000 | 0.524 | 0.277 | 0.438 | 0.272 |
| CD14 | 5.75E-07 | 2.44E-06 | 1.907 | 0.592 | 0.000 | 0.405 | 0.305 | 0.492 | 0.278 |
| TFEC | 1.00E-20 | 4.53E-20 | 0.433 | 0.933 | 0.000 | 0.503 | 0.267 | 0.332 | 0.278 |
| C3AR1 | 1.00E-20 | 4.53E-20 | 2.535 | 0.469 | 0.000 | 0.442 | 0.309 | 0.461 | 0.278 |
| FLT3 | 1.00E-20 | 4.53E-20 | 1.779 | 0.619 | 0.000 | 0.396 | 0.298 | 0.484 | 0.280 |
| HAUS2 | 1.00E-20 | 4.53E-20 | 2.736 | 0.434 | 0.000 | 0.638 | 0.286 | 0.382 | 0.285 |
| CTSW | 6.91E-06 | 2.48E-05 | 2.549 | 0.467 | 0.000 | 0.411 | 0.296 | 0.463 | 0.285 |
| LCP2 | 1.00E-20 | 4.53E-20 | 0.481 | 0.923 | 0.000 | 0.627 | 0.300 | 0.438 | 0.287 |
| SLC16A3 | 1.00E-20 | 4.53E-20 | 1.825 | 0.609 | 0.000 | 0.594 | 0.297 | 0.307 | 0.292 |
| NANOS1 | 1.00E-20 | 4.53E-20 | 2.157 | 0.540 | 0.000 | 0.595 | 0.332 | 0.489 | 0.297 |
| TCERG1L | 5.75E-07 | 2.44E-06 | 0.041 | 0.998 | 0.000 | 0.483 | 0.289 | 0.347 | 0.299 |
| NCF4 | 1.00E-20 | 4.53E-20 | 0.964 | 0.810 | 0.000 | 0.372 | 0.309 | 0.458 | 0.300 |
| PUS10 | 1.00E-20 | 4.53E-20 | 2.121 | 0.548 | 0.000 | 0.482 | 0.303 | 0.453 | 0.301 |
| LUM | 0.000546 | 0.001465 | 1.131 | 0.770 | 0.000 | 0.344 | 0.333 | 0.513 | 0.302 |
| IL1A | 4.60E-06 | 1.72E-05 | 2.973 | 0.396 | 0.000 | 0.536 | 0.266 | 0.422 | 0.302 |
| FBXL3 | 1.00E-20 | 4.53E-20 | 2.258 | 0.521 | 0.000 | 0.475 | 0.270 | 0.275 | 0.302 |
| PLAUR | 1.00E-20 | 4.53E-20 | 2.155 | 0.541 | 0.000 | 0.564 | 0.297 | 0.428 | 0.303 |
| CYP4F11 | 1.00E-20 | 4.53E-20 | 1.399 | 0.706 | 0.000 | 0.667 | 0.277 | 0.424 | 0.304 |
| SUSD1 | 1.00E-20 | 4.53E-20 | 2.498 | 0.476 | 0.000 | 0.616 | 0.310 | 0.469 | 0.304 |
| HS3ST1 | 1.00E-20 | 4.53E-20 | 1.477 | 0.688 | 0.000 | 0.804 | 0.426 | 0.607 | 0.306 |
| ARHGAP27 | 1.00E-20 | 4.53E-20 | 0.829 | 0.842 | 0.000 | 0.615 | 0.326 | 0.595 | 0.307 |
| DNASE1L2 | 1.00E-20 | 4.53E-20 | 2.403 | 0.493 | 0.000 | 0.619 | 0.310 | 0.447 | 0.311 |
| TSLP | 1.00E-20 | 4.53E-20 | 2.613 | 0.455 | 0.000 | 0.768 | 0.333 | 0.481 | 0.311 |
| BMPER | 1.00E-20 | 4.53E-20 | 2.971 | 0.396 | 0.000 | 0.841 | 0.275 | 0.263 | 0.314 |
| SLFN12 | 1.00E-20 | 4.53E-20 | 2.548 | 0.467 | 0.000 | 0.520 | 0.341 | 0.462 | 0.315 |
| SEC24A | 1.00E-20 | 4.53E-20 | 1.367 | 0.713 | 0.000 | 0.525 | 0.274 | 0.275 | 0.315 |
| FAM20A | 1.00E-20 | 4.53E-20 | 2.181 | 0.536 | 0.000 | 0.746 | 0.303 | 0.591 | 0.316 |
| HTR7 | 1.00E-20 | 4.53E-20 | 1.167 | 0.761 | 0.000 | 0.528 | 0.303 | 0.423 | 0.317 |
| HARBI1 | 1.00E-20 | 4.53E-20 | 1.512 | 0.680 | 0.000 | 0.693 | 0.312 | 0.405 | 0.317 |
| SFTPD | 1.00E-20 | 4.53E-20 | 1.313 | 0.726 | 0.000 | 0.508 | 0.317 | 0.362 | 0.318 |
| SH2D5 | 5.75E-07 | 2.44E-06 | 2.598 | 0.458 | 0.000 | 0.620 | 0.270 | 0.273 | 0.319 |
| NDE1 | 1.00E-20 | 4.53E-20 | 3.000 | 0.392 | 0.000 | 0.612 | 0.307 | 0.329 | 0.319 |
| XCR1 | 5.75E-07 | 2.44E-06 | 1.808 | 0.613 | 0.000 | 0.474 | 0.277 | 0.298 | 0.321 |
| ERC2 | 1.00E-20 | 4.53E-20 | 1.892 | 0.595 | 0.000 | 0.761 | 0.285 | 0.266 | 0.323 |
| LTO1 | 1.00E-20 | 4.53E-20 | 2.629 | 0.452 | 0.000 | 0.574 | 0.303 | 0.369 | 0.326 |
| GFOD1 | 1.00E-20 | 4.53E-20 | 0.959 | 0.811 | 0.000 | 0.372 | 0.279 | 0.530 | 0.327 |
| ZFP92 | 1.00E-20 | 4.53E-20 | 2.062 | 0.560 | 0.000 | 0.594 | 0.319 | 0.394 | 0.327 |
| MEAK7 | 1.00E-20 | 4.53E-20 | 2.724 | 0.436 | 0.000 | 0.729 | 0.335 | 0.357 | 0.328 |
| GDAP1 | 1.00E-20 | 4.53E-20 | 0.623 | 0.891 | 0.000 | 0.508 | 0.305 | 0.423 | 0.333 |
| THEMIS | 1.00E-20 | 4.53E-20 | 1.955 | 0.582 | 0.000 | 0.458 | 0.306 | 0.478 | 0.335 |
| IFITM10 | 1.00E-20 | 4.53E-20 | 0.989 | 0.804 | 0.000 | 0.664 | 0.360 | 0.520 | 0.336 |
| SLC25A19 | 1.00E-20 | 4.53E-20 | 0.755 | 0.860 | 0.000 | 0.515 | 0.316 | 0.392 | 0.338 |
| JAML | 1.00E-20 | 4.53E-20 | 2.298 | 0.513 | 0.000 | 0.390 | 0.330 | 0.522 | 0.339 |
| ACACA | 1.00E-20 | 4.53E-20 | 1.835 | 0.607 | 0.000 | 0.465 | 0.297 | 0.385 | 0.339 |
| FBXW9 | 1.00E-20 | 4.53E-20 | 0.772 | 0.856 | 0.000 | 0.499 | 0.318 | 0.402 | 0.341 |
| REEP1 | 1.00E-20 | 4.53E-20 | 1.330 | 0.722 | 0.000 | 0.490 | 0.304 | 0.340 | 0.341 |
| ADCYAP1 | 1.00E-20 | 4.53E-20 | 2.916 | 0.405 | 0.000 | 0.808 | 0.307 | 0.377 | 0.341 |
| ARHGAP9 | 1.00E-20 | 4.53E-20 | 2.591 | 0.459 | 0.000 | 0.448 | 0.343 | 0.561 | 0.341 |
| MRPS10 | 1.00E-20 | 4.53E-20 | 1.401 | 0.705 | 0.000 | 0.559 | 0.329 | 0.297 | 0.341 |
| TXNDC9 | 1.00E-20 | 4.53E-20 | 1.538 | 0.674 | 0.000 | 0.586 | 0.287 | 0.360 | 0.342 |
| TRIL | 1.00E-20 | 4.53E-20 | 1.144 | 0.766 | 0.000 | 0.431 | 0.328 | 0.520 | 0.342 |
| ATG5 | 1.00E-20 | 4.53E-20 | 2.660 | 0.447 | 0.000 | 0.511 | 0.356 | 0.401 | 0.344 |
| MON1A | 1.00E-20 | 4.53E-20 | 2.209 | 0.530 | 0.000 | 0.494 | 0.301 | 0.399 | 0.344 |
| ERP27 | 1.00E-20 | 4.53E-20 | 2.577 | 0.462 | 0.000 | 0.649 | 0.322 | 0.557 | 0.347 |
| ERI1 | 1.00E-20 | 4.53E-20 | 0.787 | 0.853 | 0.000 | 0.523 | 0.291 | 0.359 | 0.348 |
| STYK1 | 1.00E-20 | 4.53E-20 | 2.591 | 0.459 | 0.000 | 0.787 | 0.325 | 0.470 | 0.349 |
| RTN4IP1 | 1.00E-20 | 4.53E-20 | 2.865 | 0.413 | 0.000 | 0.631 | 0.340 | 0.509 | 0.353 |
| RGS18 | 1.00E-20 | 4.53E-20 | 0.265 | 0.966 | 0.000 | 0.691 | 0.370 | 0.548 | 0.354 |
| HSPA13 | 1.00E-20 | 4.53E-20 | 1.550 | 0.671 | 0.000 | 0.492 | 0.339 | 0.398 | 0.354 |
| AP1M2 | 1.00E-20 | 4.53E-20 | 2.834 | 0.418 | 0.000 | 0.552 | 0.329 | 0.332 | 0.355 |
| CCDC138 | 1.00E-20 | 4.53E-20 | 2.178 | 0.536 | 0.000 | 0.465 | 0.287 | 0.460 | 0.358 |
| PRKAR1B | 1.00E-20 | 4.53E-20 | 0.783 | 0.854 | 0.000 | 0.709 | 0.301 | 0.466 | 0.358 |
| UQCR10 | 1.00E-20 | 4.53E-20 | 1.330 | 0.722 | 0.000 | 0.644 | 0.340 | 0.436 | 0.358 |
| SPTLC1 | 1.00E-20 | 4.53E-20 | 1.395 | 0.707 | 0.000 | 0.480 | 0.317 | 0.453 | 0.362 |
| FBXL19 | 1.00E-20 | 4.53E-20 | 1.529 | 0.676 | 0.000 | 0.602 | 0.331 | 0.430 | 0.362 |
| C8orf33 | 1.00E-20 | 4.53E-20 | 1.041 | 0.791 | 0.000 | 0.724 | 0.342 | 0.418 | 0.364 |
| SAPCD2 | 1.00E-20 | 4.53E-20 | 1.020 | 0.797 | 0.000 | 0.522 | 0.296 | 0.352 | 0.364 |
| IL10RA | 1.00E-20 | 4.53E-20 | 2.749 | 0.432 | 0.000 | 0.395 | 0.344 | 0.595 | 0.369 |
| GPR171 | 1.00E-20 | 4.53E-20 | 2.333 | 0.506 | 0.000 | 0.629 | 0.387 | 0.913 | 0.370 |
| KLRC1 | 1.00E-20 | 4.53E-20 | 2.300 | 0.512 | 0.000 | 0.643 | 0.318 | 0.434 | 0.370 |
| DUSP2 | 1.00E-20 | 4.53E-20 | 2.760 | 0.430 | 0.000 | 0.614 | 0.360 | 0.467 | 0.371 |
| PPP6R1 | 1.00E-20 | 4.53E-20 | 2.259 | 0.520 | 0.000 | 0.519 | 0.312 | 0.404 | 0.374 |
| PDS5A | 1.00E-20 | 4.53E-20 | 0.371 | 0.946 | 0.000 | 0.796 | 0.338 | 0.484 | 0.377 |
| FTSJ1 | 1.00E-20 | 4.53E-20 | 1.371 | 0.712 | 0.000 | 0.537 | 0.353 | 0.404 | 0.378 |
| CCNYL1 | 1.00E-20 | 4.53E-20 | 1.111 | 0.774 | 0.000 | 0.563 | 0.374 | 0.459 | 0.378 |
| IL12B | 1.00E-20 | 4.53E-20 | 1.872 | 0.599 | 0.000 | 1.201 | 0.307 | 0.448 | 0.379 |
| PARP14 | 1.15E-06 | 4.51E-06 | 0.276 | 0.964 | 0.000 | 0.479 | 0.265 | 0.286 | 0.379 |
| CNOT9 | 1.00E-20 | 4.53E-20 | 2.811 | 0.422 | 0.000 | 0.632 | 0.378 | 0.774 | 0.384 |
| CYTIP | 1.00E-20 | 4.53E-20 | 2.258 | 0.521 | 0.000 | 0.507 | 0.383 | 0.609 | 0.385 |
| KRT24 | 1.00E-20 | 4.53E-20 | 0.641 | 0.887 | 0.000 | 0.877 | 0.321 | 0.475 | 0.392 |
| ITK | 1.00E-20 | 4.53E-20 | 1.838 | 0.607 | 0.000 | 0.669 | 0.409 | 0.704 | 0.398 |
| PIGX | 1.00E-20 | 4.53E-20 | 1.527 | 0.676 | 0.000 | 0.633 | 0.385 | 0.471 | 0.399 |
| SPINK7 | 0.001414 | 0.003514 | 2.632 | 0.452 | 0.000 | 0.602 | 0.273 | 0.451 | 0.404 |
| PTPN22 | 1.00E-20 | 4.53E-20 | 0.489 | 0.921 | 0.000 | 0.747 | 0.343 | 0.650 | 0.406 |
| API5 | 1.00E-20 | 4.53E-20 | 1.337 | 0.720 | 0.000 | 0.563 | 0.375 | 0.519 | 0.408 |
| TIMM8A | 1.00E-20 | 4.53E-20 | 0.412 | 0.938 | 0.000 | 0.709 | 0.355 | 0.463 | 0.411 |
| NUP98 | 1.00E-20 | 4.53E-20 | 2.545 | 0.467 | 0.000 | 0.554 | 0.375 | 0.404 | 0.412 |
| NAT1 | 1.00E-20 | 4.53E-20 | 0.950 | 0.813 | 0.000 | 0.561 | 0.406 | 0.414 | 0.413 |
| SPTY2D1 | 1.00E-20 | 4.53E-20 | 2.052 | 0.562 | 0.000 | 0.471 | 0.307 | 0.327 | 0.418 |
| HEATR3 | 1.00E-20 | 4.53E-20 | 0.304 | 0.959 | 0.000 | 0.875 | 0.364 | 0.579 | 0.419 |
| COX5A | 1.00E-20 | 4.53E-20 | 2.653 | 0.448 | 0.000 | 0.585 | 0.357 | 0.601 | 0.427 |
| P2RY2 | 1.00E-20 | 4.53E-20 | 0.817 | 0.845 | 0.000 | 0.880 | 0.401 | 0.949 | 0.427 |
| ST6GALNAC1 | 1.00E-20 | 4.53E-20 | 0.990 | 0.804 | 0.000 | 1.320 | 0.468 | 0.895 | 0.431 |
| OIP5 | 1.00E-20 | 4.53E-20 | 2.564 | 0.464 | 0.000 | 0.975 | 0.459 | 0.777 | 0.432 |
| GSDME | 1.00E-20 | 4.53E-20 | 1.936 | 0.586 | 0.000 | 0.652 | 0.378 | 0.541 | 0.439 |
| ERN1 | 1.00E-20 | 4.53E-20 | 1.370 | 0.713 | 0.000 | 0.449 | 0.353 | 0.487 | 0.441 |
| SUSD4 | 1.00E-20 | 4.53E-20 | 1.662 | 0.645 | 0.000 | 0.736 | 0.437 | 0.937 | 0.444 |
| PKMYT1 | 1.00E-20 | 4.53E-20 | 2.682 | 0.443 | 0.000 | 0.519 | 0.328 | 0.436 | 0.447 |
| GLT1D1 | 1.00E-20 | 4.53E-20 | 1.154 | 0.764 | 0.000 | 1.027 | 0.407 | 0.822 | 0.447 |
| CROT | 1.00E-20 | 4.53E-20 | 2.160 | 0.540 | 0.000 | 0.672 | 0.329 | 0.555 | 0.449 |
| GTF2H3 | 1.00E-20 | 4.53E-20 | 2.471 | 0.481 | 0.000 | 0.307 | 0.291 | 0.319 | 0.451 |
| VAMP3 | 1.00E-20 | 4.53E-20 | 1.477 | 0.688 | 0.000 | 0.328 | 0.288 | 0.277 | 0.452 |
| RPS21 | 2.30E-06 | 8.92E-06 | 2.779 | 0.427 | 0.000 | 0.294 | 0.270 | 0.296 | 0.453 |
| TMEM86A | 1.00E-20 | 4.53E-20 | 1.661 | 0.646 | 0.000 | 1.237 | 0.506 | 0.994 | 0.453 |
| HSD17B10 | 1.00E-20 | 4.53E-20 | 1.519 | 0.678 | 0.000 | 0.308 | 0.317 | 0.322 | 0.454 |
| LPAR3 | 1.00E-20 | 4.53E-20 | 2.549 | 0.467 | 0.000 | 0.374 | 0.314 | 0.333 | 0.454 |
| CSRNP1 | 1.00E-20 | 4.53E-20 | 2.387 | 0.496 | 0.000 | 0.405 | 0.324 | 0.413 | 0.455 |
| GNL2 | 1.00E-20 | 4.53E-20 | 1.318 | 0.725 | 0.000 | 0.293 | 0.315 | 0.287 | 0.456 |
| CYP51A1 | 1.00E-20 | 4.53E-20 | 0.455 | 0.929 | 0.000 | 0.738 | 0.353 | 0.342 | 0.456 |
| NSF | 1.00E-20 | 4.53E-20 | 1.342 | 0.719 | 0.000 | 0.330 | 0.317 | 0.343 | 0.457 |
| SYK | 1.00E-20 | 4.53E-20 | 1.751 | 0.626 | 0.000 | 0.377 | 0.318 | 0.374 | 0.457 |
| TPRKB | 1.00E-20 | 4.53E-20 | 1.055 | 0.788 | 0.000 | 0.307 | 0.310 | 0.320 | 0.459 |
| SLC25A37 | 1.00E-20 | 4.53E-20 | 0.889 | 0.828 | 0.000 | 0.293 | 0.282 | 0.345 | 0.460 |
| SMARCA5 | 1.00E-20 | 4.53E-20 | 1.833 | 0.608 | 0.000 | 0.337 | 0.320 | 0.279 | 0.461 |
| NAA50 | 1.00E-20 | 4.53E-20 | 1.511 | 0.680 | 0.000 | 0.516 | 0.335 | 0.522 | 0.462 |
| MYCBP | 1.00E-20 | 4.53E-20 | 1.619 | 0.655 | 0.000 | 0.334 | 0.282 | 0.367 | 0.462 |
| CSE1L | 1.00E-20 | 4.53E-20 | 1.165 | 0.761 | 0.000 | 0.301 | 0.300 | 0.285 | 0.463 |
| MRPL1 | 1.00E-20 | 4.53E-20 | 2.244 | 0.523 | 0.000 | 0.309 | 0.297 | 0.309 | 0.463 |
| ATG13 | 1.00E-20 | 4.53E-20 | 0.127 | 0.988 | 0.000 | 0.515 | 0.354 | 0.426 | 0.465 |
| CCDC25 | 1.00E-20 | 4.53E-20 | 0.826 | 0.843 | 0.000 | 0.320 | 0.301 | 0.329 | 0.469 |
| BTBD11 | 1.00E-20 | 4.53E-20 | 0.888 | 0.828 | 0.000 | 0.456 | 0.352 | 0.393 | 0.470 |
| NOP14 | 1.00E-20 | 4.53E-20 | 2.673 | 0.445 | 0.000 | 0.309 | 0.305 | 0.313 | 0.470 |
| ZMYND19 | 1.00E-20 | 4.53E-20 | 1.183 | 0.757 | 0.000 | 0.310 | 0.302 | 0.337 | 0.470 |
| TIPRL | 1.00E-20 | 4.53E-20 | 2.883 | 0.410 | 0.000 | 0.402 | 0.343 | 0.353 | 0.471 |
| CPSF2 | 1.00E-20 | 4.53E-20 | 1.313 | 0.726 | 0.000 | 0.557 | 0.328 | 0.359 | 0.474 |
| ATG3 | 1.00E-20 | 4.53E-20 | 0.500 | 0.919 | 0.000 | 0.301 | 0.291 | 0.286 | 0.477 |
| RPEL1 | 1.00E-20 | 4.53E-20 | 2.323 | 0.508 | 0.000 | 0.527 | 0.343 | 0.382 | 0.477 |
| JMJD7 | 1.00E-20 | 4.53E-20 | 1.854 | 0.603 | 0.000 | 0.331 | 0.309 | 0.401 | 0.479 |
| C19orf48 | 1.00E-20 | 4.53E-20 | 0.907 | 0.824 | 0.000 | 0.540 | 0.332 | 0.336 | 0.480 |
| GLRX2 | 1.00E-20 | 4.53E-20 | 0.659 | 0.883 | 0.000 | 0.370 | 0.350 | 0.335 | 0.481 |
| PWP1 | 1.00E-20 | 4.53E-20 | 1.025 | 0.795 | 0.000 | 0.304 | 0.300 | 0.292 | 0.482 |
| MCM6 | 1.00E-20 | 4.53E-20 | 2.290 | 0.514 | 0.000 | 0.425 | 0.269 | 0.382 | 0.483 |
| CDK7 | 1.00E-20 | 4.53E-20 | 0.684 | 0.877 | 0.000 | 0.302 | 0.309 | 0.308 | 0.484 |
| UBE2J1 | 1.00E-20 | 4.53E-20 | 1.258 | 0.739 | 0.000 | 0.428 | 0.352 | 0.388 | 0.488 |
| SH3GLB1 | 1.00E-20 | 4.53E-20 | 0.690 | 0.876 | 0.000 | 0.331 | 0.295 | 0.302 | 0.488 |
| KIFC1 | 1.00E-20 | 4.53E-20 | 2.431 | 0.488 | 0.000 | 0.581 | 0.311 | 0.478 | 0.494 |
| COX10 | 1.00E-20 | 4.53E-20 | 1.979 | 0.577 | 0.000 | 0.405 | 0.335 | 0.366 | 0.496 |
| SERINC5 | 1.00E-20 | 4.53E-20 | 2.096 | 0.553 | 0.000 | 0.281 | 0.287 | 0.266 | 0.499 |
| RAET1E | 1.00E-20 | 4.53E-20 | 1.627 | 0.653 | 0.000 | 0.907 | 0.439 | 0.600 | 0.500 |
| FCGR3B | 1.00E-20 | 4.53E-20 | 2.289 | 0.515 | 0.000 | 0.790 | 0.489 | 0.788 | 0.500 |
| UROS | 1.00E-20 | 4.53E-20 | 2.501 | 0.475 | 0.000 | 0.433 | 0.347 | 0.503 | 0.502 |
| FAHD1 | 1.00E-20 | 4.53E-20 | 0.507 | 0.917 | 0.000 | 0.352 | 0.332 | 0.333 | 0.504 |
| SLC30A1 | 1.00E-20 | 4.53E-20 | 2.739 | 0.434 | 0.000 | 0.479 | 0.356 | 0.598 | 0.504 |
| MASTL | 1.00E-20 | 4.53E-20 | 2.698 | 0.440 | 0.000 | 0.385 | 0.380 | 0.402 | 0.504 |
| SRP72 | 1.00E-20 | 4.53E-20 | 2.636 | 0.451 | 0.000 | 0.315 | 0.298 | 0.289 | 0.505 |
| PUSL1 | 1.00E-20 | 4.53E-20 | 2.535 | 0.469 | 0.000 | 0.593 | 0.405 | 0.593 | 0.506 |
| IL33 | 1.00E-20 | 4.53E-20 | 1.231 | 0.746 | 0.000 | 0.304 | 0.306 | 0.390 | 0.506 |
| CASP1 | 1.00E-20 | 4.53E-20 | 1.722 | 0.632 | 0.000 | 0.353 | 0.375 | 0.403 | 0.507 |
| EPRS | 1.00E-20 | 4.53E-20 | 2.720 | 0.437 | 0.000 | 0.342 | 0.307 | 0.302 | 0.509 |
| KPNA1 | 1.00E-20 | 4.53E-20 | 1.308 | 0.727 | 0.000 | 0.444 | 0.381 | 0.391 | 0.509 |
| GPR183 | 1.00E-20 | 4.53E-20 | 1.710 | 0.635 | 0.000 | 0.584 | 0.415 | 0.740 | 0.509 |
| TRMT10C | 1.00E-20 | 4.53E-20 | 0.172 | 0.982 | 0.000 | 0.337 | 0.322 | 0.336 | 0.510 |
| ALG13 | 1.00E-20 | 4.53E-20 | 2.029 | 0.566 | 0.000 | 0.357 | 0.331 | 0.385 | 0.510 |
| POLR3B | 1.00E-20 | 4.53E-20 | 1.181 | 0.758 | 0.000 | 0.431 | 0.331 | 0.368 | 0.511 |
| RTCB | 1.00E-20 | 4.53E-20 | 0.107 | 0.991 | 0.000 | 0.320 | 0.304 | 0.343 | 0.515 |
| GSKIP | 1.00E-20 | 4.53E-20 | 1.839 | 0.606 | 0.000 | 0.315 | 0.300 | 0.302 | 0.516 |
| HSPE1 | 1.00E-20 | 4.53E-20 | 2.836 | 0.418 | 0.000 | 0.303 | 0.327 | 0.282 | 0.516 |
| BDKRB2 | 1.00E-20 | 4.53E-20 | 1.348 | 0.718 | 0.000 | 0.425 | 0.352 | 0.359 | 0.517 |
| APOBEC3B | 1.00E-20 | 4.53E-20 | 2.027 | 0.567 | 0.000 | 0.779 | 0.532 | 0.649 | 0.519 |
| FAAH2 | 1.00E-20 | 4.53E-20 | 0.415 | 0.937 | 0.000 | 0.440 | 0.352 | 0.528 | 0.520 |
| C16orf70 | 1.00E-20 | 4.53E-20 | 1.961 | 0.581 | 0.000 | 0.340 | 0.348 | 0.410 | 0.526 |
| PIGF | 1.00E-20 | 4.53E-20 | 0.386 | 0.943 | 0.000 | 0.329 | 0.325 | 0.350 | 0.526 |
| PAQR4 | 1.00E-20 | 4.53E-20 | 0.225 | 0.974 | 0.000 | 0.434 | 0.322 | 0.398 | 0.526 |
| HAT1 | 1.00E-20 | 4.53E-20 | 0.797 | 0.850 | 0.000 | 0.309 | 0.300 | 0.286 | 0.528 |
| PYDC1 | 1.00E-20 | 4.53E-20 | 1.103 | 0.776 | 0.000 | 0.423 | 0.363 | 0.614 | 0.529 |
| ISG20L2 | 1.00E-20 | 4.53E-20 | 2.656 | 0.448 | 0.000 | 0.465 | 0.368 | 0.390 | 0.533 |
| RPF2 | 1.00E-20 | 4.53E-20 | 0.091 | 0.993 | 0.000 | 0.328 | 0.306 | 0.312 | 0.541 |
| NCAPD2 | 1.00E-20 | 4.53E-20 | 1.908 | 0.592 | 0.000 | 0.335 | 0.320 | 0.311 | 0.541 |
| RHOD | 1.00E-20 | 4.53E-20 | 1.063 | 0.786 | 0.000 | 0.334 | 0.295 | 0.340 | 0.545 |
| COMMD8 | 1.00E-20 | 4.53E-20 | 1.846 | 0.605 | 0.000 | 0.348 | 0.305 | 0.355 | 0.546 |
| APOBEC3A | 1.00E-20 | 4.53E-20 | 0.838 | 0.840 | 0.000 | 1.303 | 0.502 | 1.128 | 0.550 |
| NOL10 | 1.00E-20 | 4.53E-20 | 0.828 | 0.843 | 0.000 | 0.355 | 0.325 | 0.348 | 0.551 |
| PLD2 | 1.00E-20 | 4.53E-20 | 2.542 | 0.468 | 0.000 | 0.399 | 0.339 | 0.361 | 0.551 |
| FASTKD2 | 1.00E-20 | 4.53E-20 | 2.722 | 0.436 | 0.000 | 0.356 | 0.350 | 0.317 | 0.552 |
| UBE2G1 | 1.00E-20 | 4.53E-20 | 2.365 | 0.500 | 0.000 | 0.306 | 0.298 | 0.333 | 0.552 |
| ZC3H12C | 1.00E-20 | 4.53E-20 | 1.041 | 0.791 | 0.000 | 0.353 | 0.387 | 0.375 | 0.555 |
| CHIC2 | 1.00E-20 | 4.53E-20 | 2.112 | 0.549 | 0.000 | 0.400 | 0.308 | 0.341 | 0.557 |
| UTP4 | 1.00E-20 | 4.53E-20 | 2.820 | 0.420 | 0.000 | 0.343 | 0.328 | 0.319 | 0.557 |
| TDP2 | 1.00E-20 | 4.53E-20 | 2.367 | 0.500 | 0.000 | 0.351 | 0.365 | 0.340 | 0.557 |
| PEX3 | 1.00E-20 | 4.53E-20 | 1.005 | 0.800 | 0.000 | 0.439 | 0.379 | 0.349 | 0.560 |
| AMD1 | 1.00E-20 | 4.53E-20 | 2.235 | 0.525 | 0.000 | 0.284 | 0.280 | 0.272 | 0.562 |
| CD36 | 1.00E-20 | 4.53E-20 | 2.316 | 0.509 | 0.000 | 0.395 | 0.341 | 0.436 | 0.562 |
| EAF1 | 1.00E-20 | 4.53E-20 | 0.440 | 0.932 | 0.000 | 0.304 | 0.337 | 0.355 | 0.564 |
| PRMT3 | 1.00E-20 | 4.53E-20 | 2.836 | 0.418 | 0.000 | 0.419 | 0.413 | 0.393 | 0.564 |
| LYN | 1.00E-20 | 4.53E-20 | 1.087 | 0.780 | 0.000 | 0.847 | 0.467 | 0.686 | 0.569 |
| TAF13 | 1.00E-20 | 4.53E-20 | 1.132 | 0.769 | 0.000 | 0.328 | 0.346 | 0.327 | 0.571 |
| NOLC1 | 1.00E-20 | 4.53E-20 | 2.954 | 0.399 | 0.000 | 0.301 | 0.315 | 0.301 | 0.572 |
| BCL10 | 1.00E-20 | 4.53E-20 | 2.455 | 0.484 | 0.000 | 0.322 | 0.323 | 0.318 | 0.572 |
| SPATA5L1 | 1.00E-20 | 4.53E-20 | 2.527 | 0.470 | 0.000 | 0.380 | 0.367 | 0.346 | 0.573 |
| CDC42SE2 | 1.00E-20 | 4.53E-20 | 2.756 | 0.431 | 0.000 | 0.402 | 0.333 | 0.315 | 0.578 |
| TCEAL9 | 1.00E-20 | 4.53E-20 | 1.231 | 0.746 | 0.000 | 0.338 | 0.321 | 0.329 | 0.582 |
| NDUFAF2 | 1.00E-20 | 4.53E-20 | 1.989 | 0.575 | 0.000 | 0.371 | 0.368 | 0.373 | 0.583 |
| CRIPT | 1.00E-20 | 4.53E-20 | 2.717 | 0.437 | 0.000 | 0.428 | 0.351 | 0.413 | 0.585 |
| ZNRF2 | 1.00E-20 | 4.53E-20 | 1.365 | 0.714 | 0.000 | 0.353 | 0.342 | 0.363 | 0.585 |
| SMAP1 | 1.00E-20 | 4.53E-20 | 2.927 | 0.403 | 0.000 | 0.332 | 0.326 | 0.331 | 0.600 |
| TPM4 | 1.00E-20 | 4.53E-20 | 1.733 | 0.630 | 0.000 | 0.320 | 0.310 | 0.300 | 0.601 |
| STARD4 | 1.00E-20 | 4.53E-20 | 2.262 | 0.520 | 0.000 | 0.886 | 0.408 | 0.781 | 0.602 |
| MID1IP1 | 1.00E-20 | 4.53E-20 | 2.091 | 0.554 | 0.000 | 0.400 | 0.338 | 0.379 | 0.604 |
| KRTDAP | 1.00E-20 | 4.53E-20 | 1.554 | 0.670 | 0.000 | 0.293 | 0.281 | 0.272 | 0.607 |
| KRT9 | 7.94E-05 | 0.000246 | 2.047 | 0.563 | 0.000 | 0.354 | 0.332 | 0.768 | 0.607 |
| WDR77 | 1.00E-20 | 4.53E-20 | 0.047 | 0.997 | 0.000 | 0.357 | 0.371 | 0.381 | 0.608 |
| EXOSC3 | 1.00E-20 | 4.53E-20 | 0.842 | 0.839 | 0.000 | 0.401 | 0.448 | 0.401 | 0.611 |
| SQLE | 1.00E-20 | 4.53E-20 | 0.408 | 0.939 | 0.000 | 0.599 | 0.392 | 0.386 | 0.619 |
| PSMG1 | 1.00E-20 | 4.53E-20 | 0.480 | 0.923 | 0.000 | 0.367 | 0.413 | 0.344 | 0.622 |
| AREG | 1.00E-20 | 4.53E-20 | 0.354 | 0.950 | 0.000 | 0.881 | 0.534 | 0.914 | 0.635 |
| UNC93A | 1.00E-20 | 4.53E-20 | 0.852 | 0.837 | 0.000 | 0.848 | 0.372 | 0.657 | 0.639 |
| RRP15 | 1.00E-20 | 4.53E-20 | 2.387 | 0.496 | 0.000 | 0.344 | 0.365 | 0.372 | 0.646 |
| CSTA | 1.00E-20 | 4.53E-20 | 1.730 | 0.630 | 0.000 | 0.308 | 0.371 | 0.335 | 0.651 |
| LEO1 | 1.00E-20 | 4.53E-20 | 2.470 | 0.481 | 0.000 | 0.402 | 0.360 | 0.343 | 0.652 |
| SERPINB9 | 1.00E-20 | 4.53E-20 | 2.453 | 0.484 | 0.000 | 0.412 | 0.545 | 0.932 | 0.655 |
| ME1 | 1.00E-20 | 4.53E-20 | 1.598 | 0.660 | 0.000 | 0.449 | 0.366 | 0.401 | 0.660 |
| LYZ | 1.00E-20 | 4.53E-20 | 0.157 | 0.984 | 0.000 | 0.681 | 0.558 | 0.721 | 0.661 |
| UTP11 | 1.00E-20 | 4.53E-20 | 2.225 | 0.527 | 0.000 | 0.360 | 0.353 | 0.360 | 0.663 |
| SLC30A9 | 1.00E-20 | 4.53E-20 | 2.385 | 0.497 | 0.000 | 0.898 | 0.498 | 0.821 | 0.672 |
| MRPS17 | 1.00E-20 | 4.53E-20 | 2.663 | 0.446 | 0.000 | 0.489 | 0.410 | 0.429 | 0.673 |
| PSMD14 | 1.00E-20 | 4.53E-20 | 2.162 | 0.539 | 0.000 | 0.315 | 0.332 | 0.333 | 0.679 |
| SKA2 | 1.00E-20 | 4.53E-20 | 2.754 | 0.431 | 0.000 | 0.542 | 0.450 | 0.488 | 0.679 |
| C6orf62 | 1.00E-20 | 4.53E-20 | 1.312 | 0.726 | 0.000 | 0.400 | 0.318 | 0.354 | 0.689 |
| KCTD11 | 1.00E-20 | 4.53E-20 | 0.686 | 0.877 | 0.000 | 0.403 | 0.381 | 0.464 | 0.692 |
| CDT1 | 1.00E-20 | 4.53E-20 | 2.548 | 0.467 | 0.000 | 0.763 | 0.431 | 0.679 | 0.695 |
| HS3ST3A1 | 1.00E-20 | 4.53E-20 | 0.722 | 0.868 | 0.000 | 1.169 | 0.452 | 0.761 | 0.699 |
| EIF2S1 | 1.00E-20 | 4.53E-20 | 2.125 | 0.547 | 0.000 | 0.357 | 0.391 | 0.354 | 0.717 |
| PSMD11 | 1.00E-20 | 4.53E-20 | 1.453 | 0.693 | 0.000 | 0.393 | 0.369 | 0.314 | 0.721 |
| HCAR2 | 1.00E-20 | 4.53E-20 | 1.995 | 0.573 | 0.000 | 0.510 | 0.408 | 0.438 | 0.745 |
| RNASEH1 | 1.00E-20 | 4.53E-20 | 0.440 | 0.932 | 0.000 | 0.469 | 0.386 | 0.357 | 0.746 |
| XKRX | 1.00E-20 | 4.53E-20 | 2.336 | 0.506 | 0.000 | 0.580 | 0.386 | 0.581 | 0.763 |
| MED8 | 1.00E-20 | 4.53E-20 | 2.947 | 0.400 | 0.000 | 0.487 | 0.451 | 0.521 | 0.775 |
| GGCT | 1.00E-20 | 4.53E-20 | 2.681 | 0.444 | 0.000 | 0.345 | 0.378 | 0.386 | 0.804 |
| IL7R | 1.00E-20 | 4.53E-20 | 0.463 | 0.927 | 0.000 | 0.593 | 0.629 | 1.029 | 0.899 |
| MALL | 1.00E-20 | 4.53E-20 | 2.210 | 0.530 | 0.000 | 0.396 | 0.405 | 0.405 | 0.958 |
| S100A7 | 1.00E-20 | 4.53E-20 | 2.705 | 0.439 | 0.000 | 0.325 | 0.849 | 0.612 | 0.978 |
| SULT2B1 | 1.00E-20 | 4.53E-20 | 2.466 | 0.481 | 0.000 | 0.504 | 0.407 | 0.379 | 0.979 |
| MFHAS1 | 1.00E-20 | 4.53E-20 | 1.865 | 0.601 | 0.000 | 0.395 | 0.436 | 0.456 | 0.988 |
| PRDM1 | 1.00E-20 | 4.53E-20 | 1.941 | 0.585 | 0.000 | 0.453 | 0.471 | 0.579 | 1.356 |
| WFDC12 | 1.00E-20 | 4.53E-20 | 1.743 | 0.627 | 0.000 | 0.555 | 0.520 | 0.621 | 1.374 |
| C15orf48 | 1.00E-20 | 4.53E-20 | 1.275 | 0.735 | 0.000 | 0.525 | 0.481 | 0.676 | 1.451 |
| CYSRT1 | 1.00E-20 | 4.53E-20 | 2.978 | 0.395 | 0.000 | 0.526 | 0.523 | 0.616 | 1.468 |
