# Supplementary material for: The paeonol target gene autophagy-related 5 has a potential therapeutic value in psoriasis treatment
Source: PeerJ. 2021 May 25;9:e11278. doi: 10.7717/peerj.11278 (PMC8162242; doi:10.7717/peerj.11278)
Supplement: Supplemental Information 4 [file peerj-09-11278-s004.doc]

**Table S2. The functional enrichment analysis of differentially expressed genes in the protein-protein interaction network.**

| **Term** | **Count** | **PValue** | **Genes** |
| --- | --- | --- | --- |
| GO:0048285~organelle fission | 9 | 2.20E-04 | NDE1, PDS5A, NOLC1, COX10, OIP5, HAUS2, PKMYT1, SKA2, NCAPD2 |
| GO:0006461~protein complex assembly | 13 | 2.32E-04 | HSD17B10, BCL10, PSMG1, TAF13, NUP98, CSE1L, COX10, SMARCA5, GTF2H3, EPRS, VAMP3, CDK7, SYK |
| GO:0070271~protein complex biogenesis | 13 | 2.32E-04 | HSD17B10, BCL10, PSMG1, TAF13, NUP98, CSE1L, COX10, SMARCA5, GTF2H3, EPRS, VAMP3, CDK7, SYK |
| GO:0065003~macromolecular complex assembly | 14 | 8.14E-04 | HSD17B10, BCL10, PSMG1, TAF13, NUP98, CSE1L, COX10, WDR77, SMARCA5, GTF2H3, EPRS, VAMP3, CDK7, SYK |
| GO:0000280~nuclear division | 8 | 9.57E-04 | NDE1, PDS5A, NOLC1, OIP5, HAUS2, PKMYT1, SKA2, NCAPD2 |
| GO:0007067~mitosis | 8 | 9.57E-04 | NDE1, PDS5A, NOLC1, OIP5, HAUS2, PKMYT1, SKA2, NCAPD2 |
| GO:0000087~M phase of mitotic cell cycle | 8 | 1.06E-03 | NDE1, PDS5A, NOLC1, OIP5, HAUS2, PKMYT1, SKA2, NCAPD2 |
| GO:0007049~cell cycle | 15 | 1.11E-03 | PDS5A, HAUS2, PKMYT1, CDK7, NCAPD2, MCM6, CDT1, PSMD14, NDE1, NOLC1, PSMD11, OIP5, ERN1, SKA2, IL12B |
| GO:0000278~mitotic cell cycle | 10 | 1.24E-03 | NDE1, PSMD14, PDS5A, NOLC1, OIP5, PSMD11, HAUS2, PKMYT1, SKA2, NCAPD2 |
| GO:0043933~macromolecular complex subunit organization | 14 | 1.48E-03 | HSD17B10, BCL10, PSMG1, TAF13, NUP98, CSE1L, COX10, WDR77, SMARCA5, GTF2H3, EPRS, VAMP3, CDK7, SYK |
| GO:0022402~cell cycle process | 12 | 2.16E-03 | NDE1, PSMD14, PDS5A, NOLC1, OIP5, PSMD11, HAUS2, ERN1, PKMYT1, SKA2, IL12B, NCAPD2 |
| GO:0000279~M phase | 8 | 8.88E-03 | NDE1, PDS5A, NOLC1, OIP5, HAUS2, PKMYT1, SKA2, NCAPD2 |
| GO:0009057~macromolecule catabolic process | 12 | 2.23E-02 | FBXL19, PSMD14, PSMD11, UBE2G1, MED8, UBE2J1, RNASEH1, GTF2H3, CDK7, TPRKB, ATG3, ZNRF2 |
| GO:0006915~apoptosis | 10 | 2.69E-02 | BCL10, FASTKD2, CSE1L, GGCT, ATG5, SH3GLB1, ERN1, HSPE1, CASP1, API5 |
| GO:0022403~cell cycle phase | 8 | 2.78E-02 | NDE1, PDS5A, NOLC1, OIP5, HAUS2, PKMYT1, SKA2, NCAPD2 |
| GO:0012501~programmed cell death | 10 | 2.93E-02 | BCL10, FASTKD2, CSE1L, GGCT, ATG5, SH3GLB1, ERN1, HSPE1, CASP1, API5 |
| GO:0008219~cell death | 11 | 3.11E-02 | BCL10, FASTKD2, CSE1L, GGCT, ATG5, SH3GLB1, ERN1, LYZ, HSPE1, CASP1, API5 |
| GO:0016265~death | 11 | 3.24E-02 | BCL10, FASTKD2, CSE1L, GGCT, ATG5, SH3GLB1, ERN1, LYZ, HSPE1, CASP1, API5 |
| GO:0044265~cellular macromolecule catabolic process | 11 | 3.26E-02 | FBXL19, PSMD14, PSMD11, UBE2G1, MED8, UBE2J1, RNASEH1, GTF2H3, CDK7, ATG3, ZNRF2 |
| GO:0006396~RNA processing | 9 | 4.04E-02 | HSD17B10, NOLC1, PUSL1, WDR77, ERN1, EXOSC3, ERI1, FTSJ1, CPSF2 |
| GO:0042325~regulation of phosphorylation | 8 | 4.77E-02 | BCL10, LYN, ERN1, LPAR3, PKMYT1, CDK7, BDKRB2, SYK |
| hsa00100:Steroid biosynthesis | 2 | 1.40E-02 | CYP51A1, SQLE |
| hsa04662:B cell receptor signaling pathway | 3 | 1.42E-02 | BCL10, LYN, SYK |
| hsa04664:Fc epsilon RI signaling pathway | 3 | 1.51E-02 | LYN, LCP2, SYK |
| hsa04630:Jak-STAT signaling pathway | 4 | 1.57E-02 | TSLP, IL10RA, IL12B, IL7R |
| hsa04666:Fc gamma R-mediated phagocytosis | 3 | 2.05E-02 | PLD2, LYN, SYK |
| hsa04660:T cell receptor signaling pathway | 3 | 2.48E-02 | BCL10, ITK, LCP2 |
| hsa03030:DNA replication | 2 | 2.75E-02 | RNASEH1, MCM6 |
| hsa04110:Cell cycle | 3 | 3.04E-02 | PKMYT1, CDK7, MCM6 |
| hsa00190:Oxidative phosphorylation | 3 | 3.20E-02 | UQCR10, COX10, COX5A |
| hsa04650:Natural killer cell mediated cytotoxicity | 3 | 3.30E-02 | FCGR3B, LCP2, SYK |
| hsa04060:Cytokine-cytokine receptor interaction | 4 | 4.11E-02 | TSLP, IL10RA, IL12B, IL7R |
| hsa04622:RIG-I-like receptor signaling pathway | 2 | 4.70E-02 | ATG5, IL12B |

GO: Gene Ontology; KEGG: Kyoto Encyclopedia of Genes and Genomes.
