# Supplementary material for: The paeonol target gene autophagy-related 5 has a potential therapeutic value in psoriasis treatment
Source: PeerJ. 2021 May 25;9:e11278. doi: 10.7717/peerj.11278 (PMC8162242; doi:10.7717/peerj.11278)
Supplement: Supplemental Information 5 [file peerj-09-11278-s005.doc]

Table S3. The list of the 133 upregulated genes including in the protein-protein interaction network.

| **Gen symbol** |  |  |  |  |  |
| --- | --- | --- | --- | --- | --- |
| ATG5 | P2RY2 | SKA2 | ATG3 | TAF13 | EPRS |
| CDT1 | HAUS2 | COMMD8 | LCP2 | CIRH1A* | SH3GLB1 |
| PSMD14 | CCNYL1 | SMARCA5 | CDK7 | S100A7 | IL10RA |
| ITK | NAT1 | IL7R | HSD17B10 | KPNA1 | MASTL |
| GTF2H3 | SPTY2D1 | NOLC1 | CYP51A1 | NDE1 | C15orf48 |
| UQCR10 | EXOSC3 | COX5A | VAMP3 | APOBEC3A | GPR183 |
| TSLP | RNASEH1 | RRP15 | RHOD* | HCAR2 | COX10 |
| TRMT10C | PUSL1 | CPSF2* | PLD2 | EAF1 | TPRKB* |
| SYK | C19orf48 | PPP6R1 | CSE1L | FBXL19 | FAHD1 |
| SQLE | PRMT3 | NCAPD2 | OIP5 | NDUFAF2 | TFEC |
| UTP11L* | PRDM1 | SLC30A1 | POLR3B | PSMG1 | HSPA13 |
| TDP2 | PAQR4 | APOBEC3B | EIF2S1 | C6orf62 | GDAP1 |
| NOL10* | DFNA5* | TIMM8A | WDR77* | TIPRL | GLT1D1 |
| LYN | TMEM86A | CASP1 | HEATR3* | PEX3 | SULT2B1 |
| ATG13 | AREG | SPTLC1 | SLC30A9 | LYZ | PSMD11 |
| NSF | RPEL1* | ERN1 | HSPE1 | PDS5A | MCM6 |
| PWP1 | STARD4 | FAAH2* | SERPINB9 | RPMS17* | IL12B |
| ARHGAP27 | API5 | HAT1 | GGCT | PKMYT1 | NANOS1 |
| FCGR3B | BCL10 | CYTIP | RTCB | NUP98 | MED8 |
| UBE2G1 | ERI1 | ZNRF2 | ISG20L2 | CSTA | NAA50 |
| UBE2J1 | CDC42SE2 | PTPN22 | FASTKD2* | ALG13 | LEO1 |
| LPAR3 | WBP5* | FTSJ1 | BTBD11 | C16orf70 | BDKRB2 |
| ARHGAP9 |  |  |  |  |  |

*represents the genes not were included in databases including AmiGO 2 and Comparative Toxicogenomics Database.
